# Toward Practical Aluminum Organic Batteries Featuring Covalent Organic Framework

**DOI:** 10.1002/cssc.202500965

**Published:** 2025-08-05

**Authors:** Olivera Lužanin, Raquel Dantas, Ava Rajh, Urban Košir, Robert Dominko, Klemen Bučar, Matjaž Kavčič, Manuel Souto, Jan Bitenc

**Affiliations:** ^1^ Department of Materials Chemistry National Institute of Chemistry Ljubljana 1001 Slovenia; ^2^ Department of Chemistry CICECO‐Aveiro Institute of Materials University of Aveiro Aveiro 3810‐393 Portugal; ^3^ Department of Low and Medium Energy Physics Jožef Stefan Institute Ljubljana 1000 Slovenia; ^4^ Faculty of Mathematics and Physics University of Ljubljana Ljubljana 1000 Slovenia; ^5^ Faculty of Chemistry and Chemical Technology University of Ljubljana Ljubljana 1001 Slovenia; ^6^ ALISTORE – European Research Institute CNRS FR 3104 Hub de l’Energie 80039 Amiens France; ^7^ CiQUS Center for Research in Biological Chemistry and Molecular Materials Department of Physical Chemistry University of Santiago de Compostela 15782 Santiago de Compostela Spain

**Keywords:** Al‐metal batteries, covalent organic frameworks, deep eutectic solvents, organic batteries, X‐ray Raman spectroscopy

## Abstract

In recent years, Al metal organic batteries have attracted significant interest due to their promise of sustainability and high volumetric energy densities, while being based on abundant materials. However, many studies assess their performance with insufficient rigor, often emphasizing high capacity and power performance, as well as cycling stability at very high cycling rates. Herein, the feasibility of a highly stable β‐ketoenamine‐linked anthraquinone‐based covalent organic framework (DAAQ‐TFP‐COF) for rechargeable Al batteries is reassessed. First, the influence of different voltage windows on electrochemical behavior in an ionic liquid electrolyte, identifying an optimal balance between capacity and stability, is investigated. Within the optimized voltage window of 0.5 to 2.0 V, DAAQ‐TFP‐COF achieves a capacity of 113.9 mAh g^−1^ at 50 mA g^−1^. To gain deeper insight into the charge storage mechanism, surface‐ and bulk‐sensitive characterization techniques—energy‐dispersive X‐ray spectroscopy and X‐ray Raman spectroscopy—confirming monovalent AlCl_2_^+^ as the main coordination species are employed. Finally, the compatibility of DAAQ‐TFP‐COF with more cost‐effective amide‐based Al electrolytes is evaluated. In butyramide‐based electrolyte, the organic material exhibits stable performance and high Coulombic efficiency. Based on our findings, a realistic outlook on the key challenges that must be addressed to advance COF‐based electrodes for future aluminum battery applications is provided.

## Graphical abstract

This study (re)evaluates the electrochemical performance of β‐ketoenamine‐linked anthraquinone‐based covalent organic framework (DAAQ‐TFP‐COF) as a cathode for aluminum batteries. Following voltage window optimization, surface and bulk analyses identified monovalent AlCl_2_^+^ as the coordination species. The findings highlight the potential of COFs as stable cathode materials, point out the need for standardized testing protocols, and propose using alternative electrolytes that offer stable performance.

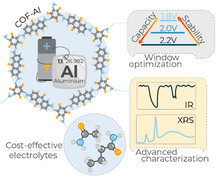

## 1 Introduction

Rechargeable aluminum metal batteries represent a promising frontier in next‐generation energy storage, offering high volumetric capacity (8040 mAh cm^−3^) and leveraging aluminum's abundance as the most prevalent metallic element in Earth's crust (8.2 wt%).^[^ ^1^ ^]^ Additionally, aluminum's well‐established production and efficient recycling process further enhance the potential sustainability of these batteries.^[^ ^1^, ^2^ ^]^ However, the high charge density of aluminum ions poses significant challenges, complicating the development of efficient electrolytes and positive electrode materials capable of reversibly storing aluminum‐based ionic species.^[^ ^3^ ^]^

The most commonly used electrolyte for aluminum‐ion batteries consists of 1‐ethyl‐3‐methylimidazolium chloride (EMIMCl) and aluminum chloride in ratio of AlCl_3_:EMIMCl > 1, which generates Al_2_Cl_7_^−^ enabling reversible Al plating.^[^ ^4^, ^5^ ^]^ Near the cathode, the dissociation of Al_2_Cl_7_^−^ leads to the formation of thermodynamically unstable AlCl^2+^ and AlCl_2_^+^ species, which can coordinate reduced functional groups in organic materials (Table S2, Supporting Information).^[^ ^6^ ^]^ Redox‐active organic materials are well‐suited for accommodating chloroaluminate ionic species as they are typically characterized by softer and adaptable structures, which facilitate reversible ion insertion and extraction. At the active material level, aluminum organic batteries could theoretically achieve specific energy densities of up to 200 Wh kg^−1^, combining high performance with economic viability, the potential recently demonstrated on poly(phenanthrene quinone) (PFQ).^[^ ^7^ ^]^ However, despite their promise, many organic electrodes suffer from poor cycling stability due to the high solubility in the electrolyte, both in pristine and reduced states.^[^ ^8^ ^]^

A potential strategy to mitigate this issue is the use of covalent organic frameworks (COFs), a subclass of redox‐active organic materials with robust and well‐defined crystalline porous structures. COFs offer high cycling stability, chemical and structural versatility, and the ability to incorporate multiple redox‐active centers tailored for specific applications. These properties have made COFs attractive hosts for a variety of post‐Li battery chemistries, including lithium, magnesium, and aluminum.^[^ ^9^, ^10^, ^11^, ^12^, ^13^, ^14^, ^15^ ^]^ Compared to commercial inorganic electrodes, such as nickel‐manganese‐cobalt oxides (NMC), redox‐active COFs eliminate the need for transition metal mining and extraction, making them a potentially more sustainable and cost‐effective alternative.^[^ ^16^ ^]^

Among COFs, one of the most widely studied compounds (DAAQ‐TFP‐COF) combines diaminoanthraquinone (DAAQ) moieties with two carbonyl groups and 1,3,5‐triformylphloroglucinol (TFP) units linked by β‐ketoenamines (Figure S1, Supporting Information).^[^ ^17^ ^]^ DAAQ‐TFP‐COF has demonstrated reversible activity in lithium, magnesium, and aluminum batteries, serving as a host for both monovalent and multivalent cations.^[^ ^18^, ^19^ ^]^ However, research reports on redox‐active organic materials often overestimate electrochemical performance by cycling outside the electrolyte's stability window and using very low electrode loadings (>1 mg cm^−2^). Furthermore, the study of the electrochemical mechanism relies primarily on surface‐sensitive techniques, such as X‐ray photoelectron spectroscopy and energy‐dispersive X‐ray spectroscopy (EDX), which may not provide a comprehensive understanding of the mechanism and can be influenced by surface‐specific effects.^[^ ^6^, ^20^ ^]^

Given the growing interest in Al metal‐organic batteries, it is essential to establish fair and reproducible evaluation criteria for different active materials. In this study, we conduct a comprehensive electrochemical evaluation of the DAAQ‐TFP‐COF electrode in a benchmark EMIMCl and AlCl_3_ (1.5 molar ratio) ionic liquid electrolyte. We assess its performance across three different upper voltage cutoffs and characterize the coordinating species using both surface‐sensitive technique EDX and bulk‐sensitive X‐ray Raman scattering (XRS), a technique specifically suited for investigation of light elements such as C, N, and O within the bulk. Furthermore, we investigate COF's performance in cost‐effective, amide‐based Al electrolytes, providing a thorough overview of its viability for next‐generation rechargeable aluminum batteries.

## 2 Results and Discussion

### 2.1 Effect of Cycling Conditions on the Electrochemical Response of COF

The carbonyl‐rich COF was synthesized by condensation reaction of 2,6‐diaminoanthraquinone and 1,3,5‐triformylphloroglucinol by adapting the reported procedure (Figure S1, Supporting Information).^[^ ^17^ ^]^ Material properties of DAAQ‐TFP‐COF were evaluated through a variety of characterization techniques (Figure S2‐S8, and Table S1, Supporting Information). The theoretical powder X‐ray diffraction (PXRD) pattern based on the eclipsed AA stacking model showed good agreement with the PXRD experimental data, and a satisfactory Pawley refinement of DAAQ‐TFP‐COF was achieved (Figure S2, Supporting Information). Infrared (IR) and ^13^C solid‐state cross‐polarization magic angle spinning nuclear magnetic resonance (CPMAS NMR) (Figure S3‐S4, Supporting Information) spectroscopies confirmed the formation of the β‐ketoenamine‐linked COF. Nitrogen adsorption‐desorption isotherm of activated DAAQ‐TFP‐COF was measured at 77 K (Figure S6, Supporting Information), and the pore size distribution was determined using the nonlocal density functional theory method (Figure S7, Supporting Information), revealing a pore width of 20 Å. Scanning electron microscopy (SEM) analysis revealed that DAAQ‐TFP‐COF consists of agglomerated particles with a size in the range of several 100 nm (Figure S8, Supporting Information). Electrodes were prepared using DAAQ‐TFP‐COF as the active material, following the procedure described in the Experimental Section, with a final active material loading of ≈2 mg cm^−2^. While not exceptionally high, this loading is notably higher than those used in the previous DAAQ‐TFP‐COF/Al report.^[^ ^18^ ^]^ Based on previous studies involving organic/Al systems, we selected an electrolyte composed of EMIMCl and AlCl_3_ in a molar ratio of 1:1.5 (denoted as 1.5EA). Linear sweep voltammetry with a molybdenum working electrode at a sweep rate of 0.1 mV s^−1^ indicated electrolyte oxidative stability up to 2.4 V (Figure S9, Supporting Information), while galvanostatic metal plating/stripping test at 0.1 mA s^−1^ had an average aluminum plating/stripping efficiency of 97%.

However, it is crucial to note that electrolyte stability estimates obtained using low surface‐area electrodes (such as molybdenum metal electrode) may not accurately reflect real battery conditions, where electrodes typically exhibit significantly higher specific surface areas. For instance, DAAQ‐TFP‐COF has a surface area of 720 m^2^ g^−1^, as calculated from N_2_ adsorption isotherm using the BETSI method^[^ ^21^ ^]^ (Brunauer–Emmett–Teller Surface Identification) (Figure S6, Supporting Information), while Printex XE2 carbon black has a surface area of ≈950 m^2^ g^−1^.^[^ ^20^ ^]^ To assess the true stability of the system under realistic conditions, we examined the performance of DAAQ‐TFP‐COF in 1.5EA across three different voltage windows at a low current density of 50 mA g^−1^.

**Figure 1:**
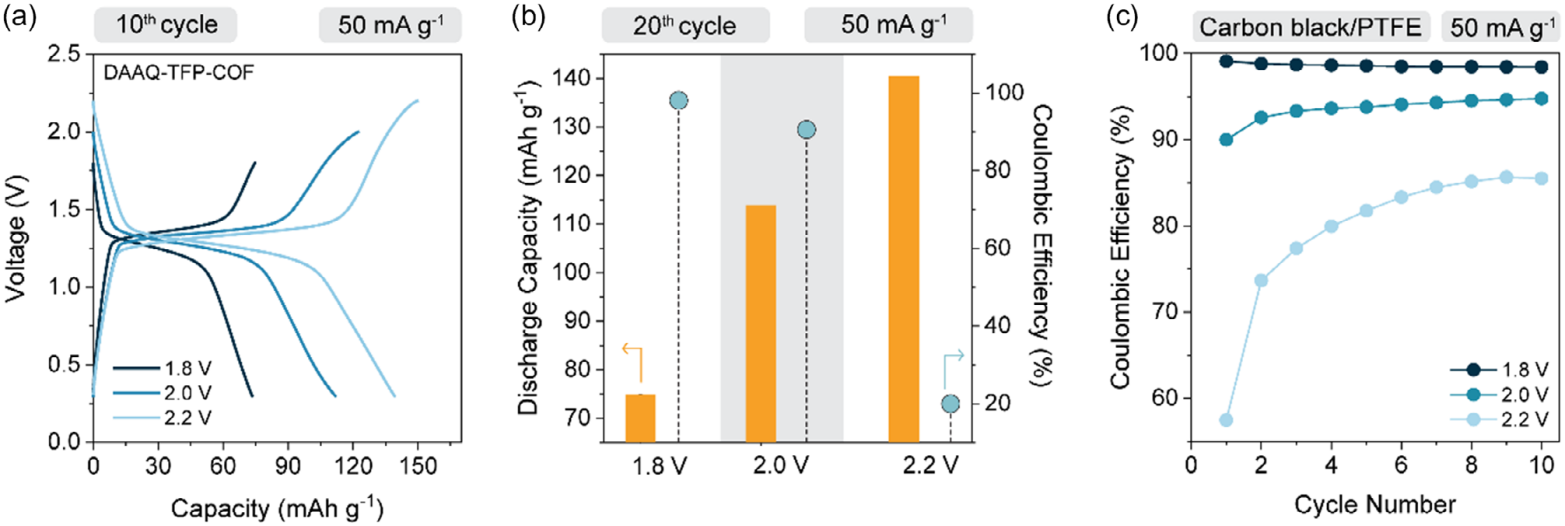
Evaluation of DAAQ‐TFP‐COF in 1.5EA electrolyte under different cycling conditions. a) Comparison of the 10th galvanostatic cycle of DAAQ‐TFP‐COF electrode at 50 mA g^−1^ in three voltage windows—0.3–1.8 V, 0.3–2.0 V, and 0.3–2.2 V. b) Discharge capacity obtained under the same condition in 20th cycle (orange bar) and Coulombic efficiency in the same cycle (blue dot) as a function of upper voltage cutoff. c) Coulombic efficiencies of carbon black:PTFE (polytetrafluoroethylene) electrode without active material, cycled in different voltage windows with increasing upper voltage cutoff. Galvanostatic profiles are available in Figure S10, Supporting Information.

Galvanostatic charge/discharge cycling was conducted with upper voltage cutoffs of 1.8, 2.0, and 2.2 V. As shown in **Figure** **1**a, the 10th cycle voltage profiles exhibited characteristic sloping curves between 1.3 and 1.1 V indicative of anthraquinone redox activity, with average discharge voltages around 1.2 V. As expected, capacity increased with a wider voltage window, while narrowest window yielded the lowest capacity. Comparing the capacities obtained in the 10th and 20th cycle, the values in the narrowest window remained stable, in both cases at around 75 mAh g^−1^. Extending the upper cutoff to 2.0 V led to a ≈65% capacity increase compared to a 1.8 V cutoff, delivering 113.9 mAh g^−1^ in both cycles. At 2.2 V, the discharge capacity approached the theoretical value, registering at 139 mAh g^−1^. However, Coulombic efficiencies decrease significantly with increasing upper cutoff voltage. While it was relatively high at 1.8 V and 2.0 V (97% and 91% on average, respectively), the irreversible overcharge at 2.2 V cutoff increased significantly after the 15th cycle (Figure S10d, Supporting Information), likely due to electrolyte decomposition and the associated side reactions during charge, leading to only 20% Coulombic efficiency (Figure 1b).

To further investigate these effects, we conducted control experiments using electrodes composed solely of Printex XE2 carbon black and PTFE binder. As anticipated, Coulombic efficiency remained below 90% at the 2.2 V cutoff (Figure 1c), confirming the impact of electrolyte instability. It is important to note that the capacity of Printex XE2 in a voltage window of around 2 V is significantly larger (≈60 mAh g^−1^ for 1.5EA electrolyte) than for typical Li‐ion electrolytes (≈30 mAh g^−1^ for LiTFSI 1,3‐dioxolane:dimethoxyethane = 1:1 electrolyte).^[^ ^19^ ^]^ Upon cycling of active material at higher current density (200 mA g^−1^) in the voltage window up to 2.2 V, efficiency improved, averaging ≈93% over 50 cycles (Figure S11, Supporting Information). While high‐rate cycling could be important for practical applications as it enables high‐power battery operation, the assessment of performance at lower currents is equally, if not more important, yet it remains overlooked in current literature reports. Low‐rate testing helps to identify potential side reactions and instabilities in both the electrolyte and active material, providing key insight into the long‐term cell behavior that determines its practical viability.

Based on the trade‐off between recorded capacity and Coulombic efficiency, all further experiments in 1.5EA electrolyte were conducted with an upper voltage cutoff of 2.0 V. During the long‐term cycling assessment of DAAQ‐TFP‐COF, a repeatable gradual increase in capacity was observed over the first 10 cycles, attributed to an activation process involving electrolyte uptake.^[^ ^18^, ^22^ ^]^ This activation was confirmed by cyclic voltammetry, where both the reduction and oxidation peaks, located at 1.27 and 1.37 V, respectively, exhibited a progressive increase in intensity during the initial cycles (Figure S12, Supporting Information). Interestingly, the activation remained evident even when an electrochemically assisted swelling strategy was applied (Figure S13, Supporting Information),^[^ ^22^ ^]^ suggesting that the electrolyte uptake into COF is relatively slow.

**Figure 2:**
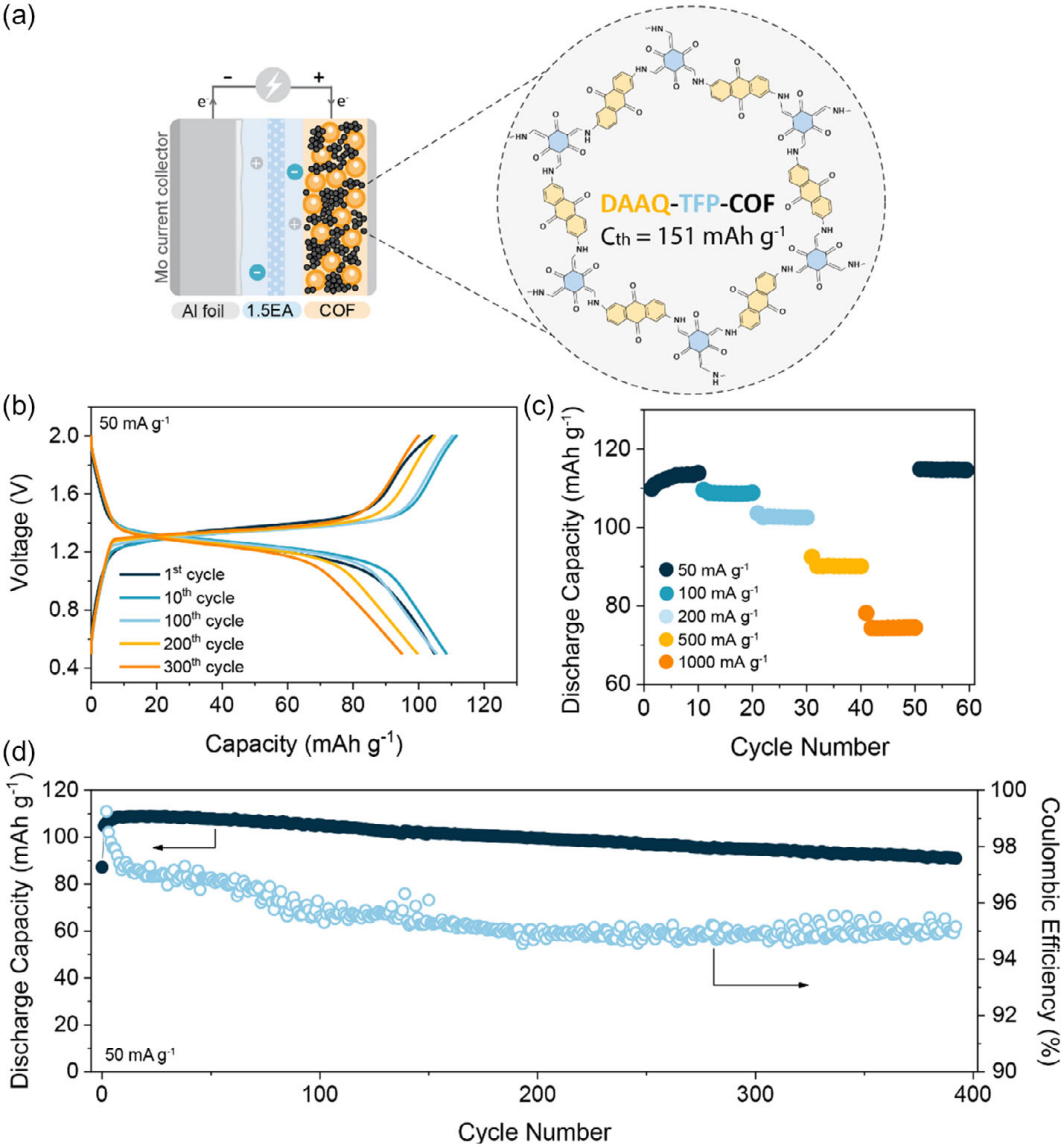
Evaluation of DAAQ‐TFP‐COF active material in aluminum battery in optimized voltage window from 0.5 to 2.0 V. a) Schematic design of aluminum metal organic cathode and structure of DAAQ‐TFP‐COF with corresponding theoretical capacity. b) Selected charge/discharge profiles obtained at 50 mA g^−1^ during long‐term cycling. c) Rate capability performance in the range from 50 to 1000 mA g^−1^. d) Long‐term stability over 400 cycles at 50 mA g^−1^ with corresponding Coulombic efficiency.

Following the initial activation phase, the COF‐based organic electrode demonstrated stable cycling, retaining 87% of its capacity after 400 charge/discharge cycles with an average Coulombic efficiency of 95% (**Figure** **2**d). At currents as high as 1 A g^−1^, the COF achieved reversible capacity of 75 mAh g^−1^ (Figure 2c). After accounting for the contribution from carbon black capacitance, the active material capacity was 63 mAh g^−1^, corresponding to 42% of theoretical capacity utilization (Table S3, Supporting Information). Based on previous reports in Li, Mg, and Al batteries,^[^ ^18^, ^19^ ^]^ COF cathode likely supports even higher current densities. However, in a two‐electrode setup, performance at currents higher than 1 A g^−1^ is constrained by processes occurring at the Al metal anode, making it difficult to isolate cathode performance under such conditions. Nevertheless, at all explored current densities, DAAQ‐TFP‐COF exhibited stable, reversible behavior with high‐capacity retention.

### 2.2 Redox Mechanism and Ion Speciation

**Figure 3:**
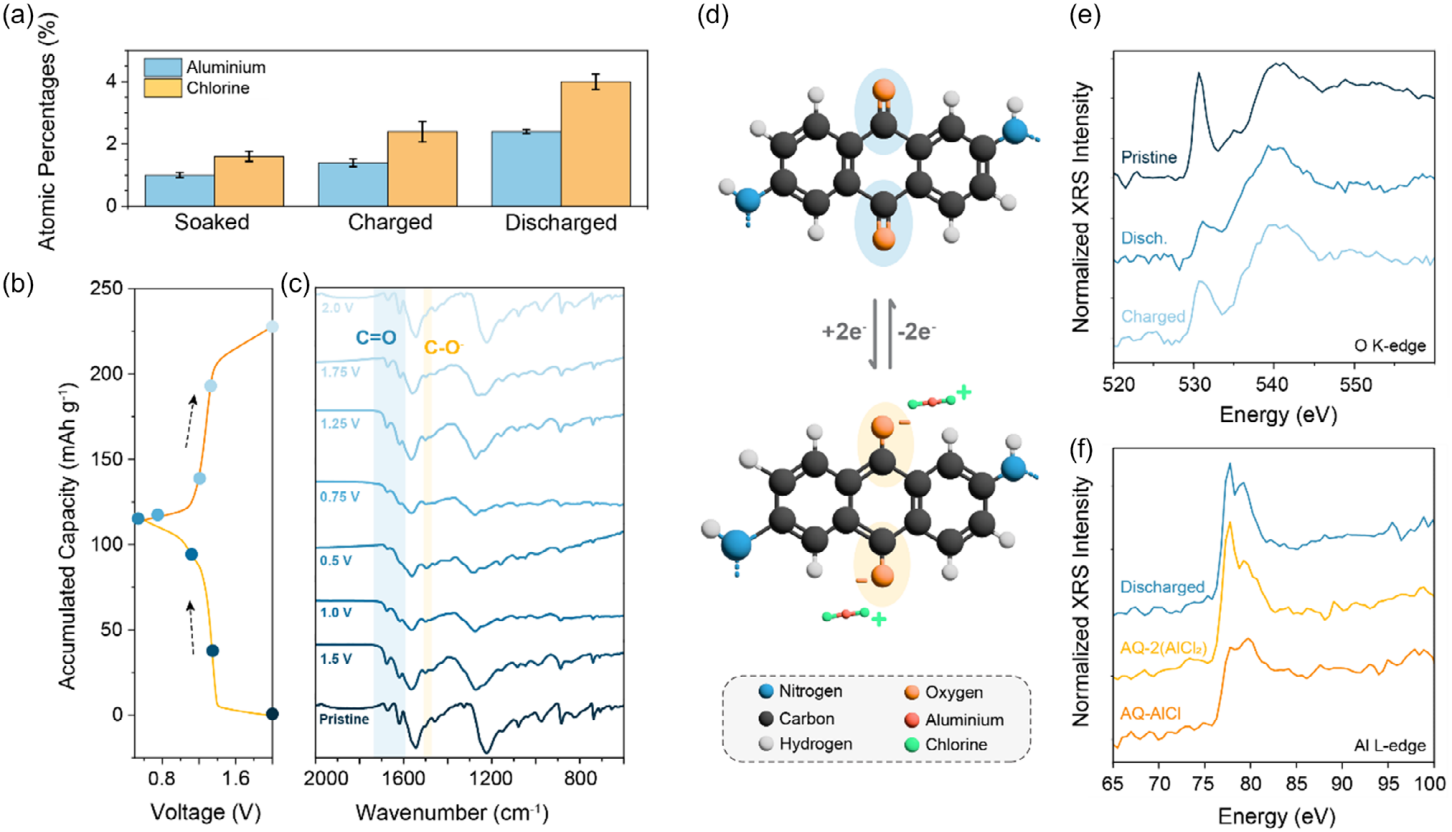
*Ex situ* characterization of DAAQ‐TFP‐COF electrodes. a) Atomic percentages of Al (blue) and Cl (orange) recorded in soaked, charged, and discharged electrodes as determined by EDX analysis. b) Galvanostatic charge/discharge profile with labeled voltages at which cells were stopped, and IR spectra of electrodes were obtained. c) IR spectra of pristine (uncycled) and *ex situ* DAAQ‐TFP‐COF electrodes obtained at different points of the discharge/charge cycle. d) Mechanism illustration of the DAAQ electroactive unit within COF. e) Oxygen K‐edge XRS spectra of pristine, fully discharged, and charged DAAQ‐TFP‐COF electrodes. f) Aluminum L_2,3_‐edge XRS spectra of discharged DAAQ‐TFP‐COF electrode and two model compounds—AQ‐2(AlCl_2_) and AQ‐AlCl, illustrating two different chloroaluminate species coordination scenarios of AQ redox group in discharged state.

Based on the observed average discharge voltage (≈1.2 V), the shape of charge/discharge curves, and the obtained capacities, it is reasonable to assume that, within the selected voltage window, only the carbonyl groups of the DAAQ unit are electrochemically active. This assumption aligns with previous reports on the same compound in lithium and magnesium cells.^[^ ^19^ ^]^ The pristine electrode spectrum, as shown in **Figure** **3**c, closely resembles that of a pure powder, indicating that the addition of carbon black and PTFE binder does not affect the main peaks of the polymer (Figure S14, Supporting Information). Distinct carbonyl stretching peaks at 1618 and 1670 cm^−1^ can be observed in the pristine electrode, both stemming from C=O groups from DAAQ. In contrast, the spectrum of the pristine linker, TFP, shows a carbonyl peak at 1658 cm^−1^ (Figure S3, Supporting Information). After incorporation into COF, this peak shifts to the lower wavenumbers and merges with C=C stretching, resulting in one broad band in the spectra of the pristine COF electrode at 1546 cm^−1^.

Upon gradual discharge down to 0.5 V (Figure 3b), carbonyl peaks diminish in intensity, which corresponds with the recorded incomplete capacity utilization (≈66%). Accompanying this increase in intensity, the new band appears at 1494 cm^−1^ corresponding to the formation of C—O^−^. Additionally, the C—N band, located at 1224 cm^−1^ in the pristine electrode, shows changes in both shape and intensity, with an upshift to 1282 cm^−1^ observed across all cycled electrodes. Even after soaking and subsequent washing, the peak demonstrates a redshift. These observations suggest that the changes may be attributed to interactions between residual electrolyte and the DAAQ‐TFP‐COF. When the electrode is recharged back to 2.0 V, the carbonyl groups regain their initial intensity while the C—O^−^ band disappears, indicating excellent reversibility of the electrochemical reaction. Mechanism of the repeating unit is illustrated in Figure 3d.

While the IR spectra align with the observed electrochemical behavior, the question of which ion species coordinates with the reduced carbonyl groups remains. To identify the charge carrier, we combined surface‐sensitive EDX and bulk‐sensitive XRS. EDX served as a preliminary tool to assess the ratio between Al and Cl species in the reduced electrode. To ensure proper electrode washing, we first evaluated the Cl and Al content in electrodes soaked in 1.5EA overnight, then washed with tetrahydrofuran (THF), which proved to be the most effective solvent for removing residual ionic liquid (see Experimental Section for details).^[^ ^23^ ^]^ In the soaked electrode, both Al and Cl were detected. Extending the THF wash time from 10 min to 1 h did not significantly reduce the presence of these elements, suggesting that small amounts of ionic liquid remain embedded in the electrode. A similar Cl:Al ratio of 1.7 was observed in the fully charged electrode, where no ionic species are expected to coordinate with the carbonyl groups. This further supports the conclusion that some residual electrolyte persists within the electrode.

Based on atomic ratios (Figure 3a), we can estimate the charge carrier involved in coordination. In the fully discharged electrode, the Cl:Al ratio is around 1.7, suggesting predominant coordination with AlCl_2_^+^ species. However, when examining the Cl:Al ratios in other cases, such as soaked and charged electrodes (Table S4, Supporting Information), the Cl to Al ratio is also close to 1.7, pointing to limited reliability of ionic species determination by EDX. In theory, the Cl:Al ratio in these cases should be closer to 3.7, aligning with values expected in the 1.5EA electrolyte. Nonetheless, if we assume monovalent coordination, it has direct implications for the energy density of the potential battery employing DAAQ‐TFP‐COF active material with an EA electrolyte. Considering the estimated coordination with monovalent species, an average discharge potential of 1.2 V, and the recorded cathode capacity of 113.9 mAh g^−1^, the approximate specific energy density of the Al‐COF cell could reach 62 Wh kg^−1^, based only on the active material. If coordination occurred primarily with divalent AlCl^2+^ species, reducing Cl:Al ratio closer to 1, the estimated specific energy density would nearly double to ≈103 Wh kg^−1^, displaying a key influence of the chloroaluminate species nature on the energy density of a practical Al battery (calculation details available in Section 6, Supporting Information).

It is important to note that while EDX provides an approximation of the ionic species involved in electrochemical storage, it remains a surface‐sensitive technique and may not offer a fully reliable charge carrier determination in the bulk. Additionally, EDX is not well‐suited for detecting light elements, which predominantly constitute organic electrode materials. Thus, complementary bulk‐sensitive techniques, such as XRS, are necessary for a more comprehensive analysis.

To further investigate the ion speciation and confirm the electrochemical mechanism, we employed XRS. XRS is a photon‐in/photon‐out characterization technique based on the inelastic scattering of high‐energy photons on the core electrons. In the process, a part of the photon's energy is transferred to the electron, promoting it into an unoccupied state. By monitoring the energy loss of the scattered photons, the absorption edge of an element can be recorded. This signal provides element‐specific information about the local electronic structure and symmetry around target atoms. What makes this technique appealing for studying redox‐active organic materials is that it enables the study of the absorption edges of low Z‐elements in the soft X‐ray range—such as C, N, and O—with bulk sensitivity provided by the high penetration depth of the incident hard X‐ray probe. It has already been successfully applied to measure the oxygen K‐edge in carbonyl‐containing compounds coupled with Li, Mg, and Al charge carriers.^[^ ^24^ ^]^

As a starting point, O K‐edge XRS spectra were recorded of pristine, fully discharged, and charged DAAQ‐TFP‐COF electrodes (Figure 3e). A pronounced sharp resonance at 531 eV, characteristic for the carbonyl bond^[^ ^24^ ^]^ has been clearly observed in the measured XRS spectrum of the pristine electrode. Upon discharge, the intensity of this resonance decreases to 20%, consistent with high but incomplete capacity utilization (measured at 66% after capacitance reduction, Table S3, Supporting Information). After recharge, it does not regain the intensity of the pristine electrode, indicating certain difference between the charged and pristine state of active material (Figure S15, Supporting Information), consistent with the previous XRS study on AQ‐based polymer.^[^ ^24^ ^]^ The change in C=O resonance intensity confirms the evolution of aromatic carbonyl groups during cycling.

To evaluate the Al coordination species, we synthesized model compounds, namely, reduced anthraquinone (AQ) salts, AQ‐2(AlCl_2_) and AQ‐AlCl (Figure S16, Supporting Information). Anthraquinone, as a small model molecule, cannot fully represent the complexity of the COF structure. However, it enables the synthesis of reduced analogues mimicking the active AQ group of the DAAQ‐TFP‐COF material. Anthraquinone was chemically reduced to 1,10‐dihydroxyanthracene and subsequently reacted with Al organometallic compounds to yield two standards (Section 5, Supporting Information). These two model compounds represent two cases of coordination, with either two monovalent AlCl_2_^+^ or one bivalent AlCl^2+^ per AQ unit. The Al L_2,3_ edge XRS spectra of both model compounds were recorded and compared qualitatively to the spectrum recorded on the discharged DAAQ‐TFP‐COF electrode (Figure 3f).

When comparing the discharged DAAQ‐Al‐COF electrode spectrum to the corresponding spectra of both standards, it closely resembles the AQ‐2(AlCl_2_) case, suggesting coordination with monovalent AlCl_2_^+^ species. However, besides Al bound to the oxygen in a discharged state, part of the signal can also be attributed to the residual electrolyte, and separating these components proved difficult. Given the significant reduction in oxygen K‐edge intensity and the weak Al signal in the discharged electrode, it is likely that the proportion of electrolyte in the sample is small. Therefore, a major contribution to the L‐edge Al spectrum of discharged COF appears to stem from AlCl_2_^+^ species bound to the C—O^−^ bond. The results obtained from the advanced XRS technique are consistent with those observed in the IR and EDX analyses, though they remain qualitative.

### 2.3 Electrochemical Performance in More Cost‐Effective Electrolytes

**Figure 4:**
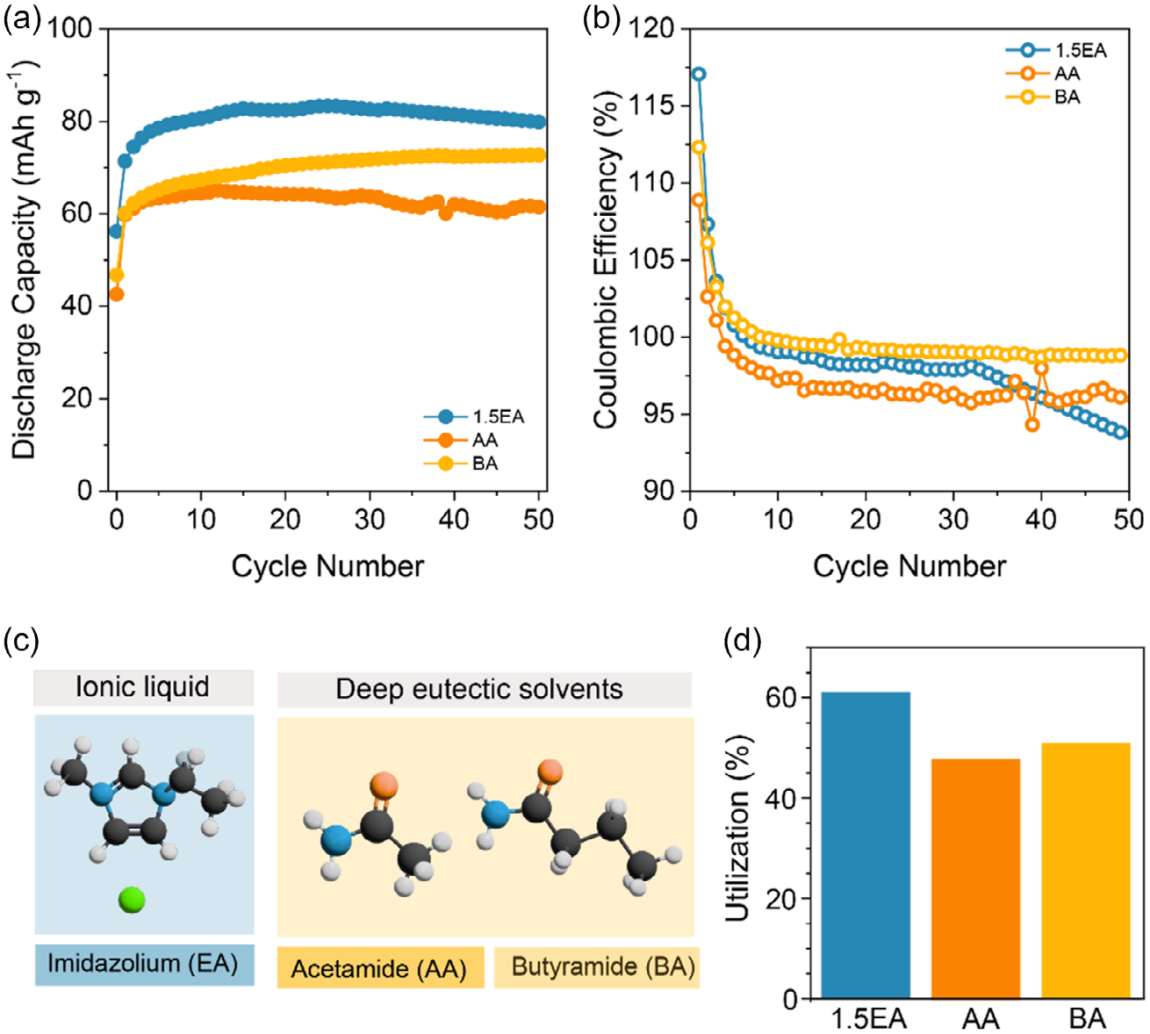
Electrochemical performance of DAAQ‐TFP‐COF cathode in amide‐based Al electrolytes compared to 1.5EMIMCl in the voltage window up to 1.8 V at 50 mA g^−1^. a) Discharge capacity evolution over 50 cycles in 1.5EA (blue), AA (dark orange), and BA (light orange). b) Coulombic efficiency over 50 cycles. c) Structures of EMIMCl, AA, and BA. d) Capacity utilization in selected electrolytes, calculated as the highest discharge capacity obtained at 50 mA g^−1^, divided by theoretical capacity (151 mAh g^−1^).

Although EMIMCl‐based electrolyte exhibits good compatibility with DAAQ‐TFP‐COF and has been used with most of the organic electrodes reported to date.^[^ ^6^ ^]^ High cost of the electrolyte (making up around 28% of the total Al battery price according to the techno‐economic modelling^[^ ^7^ ^]^) raises concerns about the practical applicability of Al‐based organic batteries using this electrolyte. To gain a more comprehensive understanding of the feasibility of Al batteries with DAAQ‐TFP‐COF cathode, we assessed its performance in alternative cost‐effective electrolytes (Table S5, Supporting Information). These include deep eutectic solvent electrolytes based on acetamide (AA) and butyramide (BA), which have been shown to reversibly plate Al at room temperature^[^ ^25^ ^]^ and demonstrate good compatibility with carbonyl‐based organic electrodes.^[^ ^26^ ^]^ The structures of the selected solvents are shown in **Figure** **4**c.

For a fair comparison, electrochemical cycling was performed with an upper voltage cutoff of 1.8 V, considering the lower oxidative stability of amide‐based electrolytes (Figure S17, Supporting Information). The voltage profiles of DAAQ‐TFP‐COF in alternative electrolytes closely resemble those recorded in 1.5EA, exhibiting the characteristic sloping curves of anthraquinone‐based active unit (Figure S18, Supporting Information). However, the average overpotentials in amide‐based electrolytes are ≈10 mV higher compared to 1.5EA, registering at around 85 mV. These values were measured in half‐cells with Al metal, meaning the contributions from both metal anode and organic cathode cannot be individually distinguished. Among the tested electrolytes, 1.5EA yielded the highest capacity (85 mAh g^−1^), while the lowest capacity (62 mAh g^−1^) was recorded in AA (Figure 4a). The BA electrolyte, in contrast, demonstrated excellent cycling stability, with the gradual capacity increase suggestive of an activation process. Additionally, its Coulombic efficiency remained consistently high, surpassing that of AA and 1.5EA, averaging 99% over 50 cycles (Figure 4b).

The reversibility of the electrochemical mechanism in both amide‐based electrolytes was investigated using IR spectroscopy (Figure S19, Supporting Information). In both cases, the spectra of discharged electrodes exhibit strong peaks associated with amides in the high wavenumber region (3000–2800 cm^−1^), corresponding to N—H stretching.^[^ ^27^, ^28^ ^]^ Since amides also contain a carbonyl group, their peaks overlap with the C=O region of interest in DAAQ, preventing a clear observation of carbonyl group intensity changes. Additionally, amides exhibit a band around 1490 cm^−1^, which overlaps with the enolate stretching peak at ≈1500 cm^−1^. The presence of strong N—H stretching in the IR spectra of both AA and BA suggests coordination with the amide‐based Al complexes. This is further supported by EDX analysis (Table S6, Supporting Information), which confirms nitrogen detection in the discharged electrodes of both AA and BA. Upon recharging, the bands stemming from amides diminish in intensity, while the C=O stretching bands from DAAQ become clearly observable at 1676 and 1623 cm^−1^ in both cases. This confirms that, upon recharging, the carbonyl groups are successfully oxidized back to C=O.

DAAQ‐TFP‐COF exhibited good electrochemical performance in both amide‐based electrolytes, comparable to 1.5EA. The slightly lower capacities observed are attributed to the limited voltage window, resulting from the previously mentioned low oxidative stability of amides. Nevertheless, the COF outperforms the linear anthraquinone‐based polymer in both AA and BA, particularly in terms of cycling stability, underscoring the potential of COFs for future investigation.^[^ ^26^ ^]^

## 3 Conclusion

In this study, we provided a fresh perspective on the electrochemical evaluation of DAAQ‐TFP‐COF as a cathode for rechargeable aluminum batteries. By defining 2.0 V as the optimal upper cutoff voltage, we achieved a capacity of 113.9 mAh g^−1^ at 50 mA g^−1^, with 87% capacity retention after 400 cycles. Surface characterization techniques were complemented by XRS, which allows bulk investigation of light elements. The combined characterization approach confirmed monovalent AlCl_2_^+^ as the primary coordination species, based on *ex situ* analysis of electrodes and model AQ‐based compounds.

Our findings clearly attribute the electrochemical activity to the DAAQ carbonyl groups, in contrast to previous reports suggesting the involvement of the β‐ketoenamine linkages.^[^ ^18^ ^]^ The overestimation of active material capacity in previous reports can be attributed to the high capacitance response of carbon black in aluminum electrolytes, as well as the use of overextended voltage windows and low active material loadings. This highlights the need for more reliable electrochemical testing protocols and a comprehensive characterization approach that combines both surface and bulk analysis techniques.^[^ ^20^ ^]^

In pursuit of more cost‐effective aluminum batteries, we explored aluminum amide‐based electrolytes as an alternative to conventional ionic liquids. A butyramide‐based electrolyte demonstrated excellent cycling stability, maintaining 99% Coulombic efficiency, thereby highlighting the potential of alternative aluminum electrolytes. While DAAQ‐TFP‐COF exhibits stable cycling, reversible charge storage, and broad electrochemical compatibility with Al electrolytes, the energy density of an Al metal/DAAQ‐TFP‐COF cell remains limited to 62 Wh kg^−1^. This represents a clear challenge for future research focused on enhancing the energy density of Al batteries. Although modifying the electroactive organic groups could improve the cathode's operating voltage and capacity, achieving selective coordination of AlCl_2_^+^ species remains a greater challenge. Addressing this issue will likely require modification of both the cathode material and the electrolyte composition.

## 4 Experimental Section

4.1

4.1.1

##### COF Synthesis and Electrode Preparation

All reagents were of high purity grade and were purchased from Sigma Aldrich or TCI. All reagents were used as received. DAAQ‐TFP‐COF was synthesized adapting a reported procedure.^[^ ^17^ ^]^ Physicochemical characterization of DAAQ‐TFP‐COF was performed by PXRD, IR spectroscopy, thermogravimetric analysis, N_2_ adsorption isotherms, CPMAS NMR, and SEM. Details of synthesis, chemicals, and structural characterization can be found in Supporting Information.

Electrodes were prepared by mixing DAAQ‐TFP‐COF powder with Printex XE2 carbon black (Degussa) and PTFE binder (Sigma‐Aldrich, 60% water dispersion) in a weight ratio of 6:3:1, with the addition of isopropanol to ensure good mixing. Dispersion was subjected to planetary ball milling (Retsch PM100, 300 rpm, 30 min). The obtained composite was rolled into a thin film between a glass plate and a parchment paper. Self‐standing 12 mm electrodes were cut, with the average mass loading of active material of around 2 mg cm^−2^ and electrode thickness of 60 μm. Prior to cell assembly, electrodes were dried at 50 °C in a vacuum oven overnight.

##### Electrolyte Preparation and Electrochemical Measurements

For electrolyte preparation, aluminum chloride (Sigma‐Aldrich, 99.99%) and 1‐ethyl‐methylimidazolium chloride (Acros, 97%) were used as received. Commercial amides (Sigma‐Aldrich, AA ≥ 99.0%, BA ≥ 98.0%) were dried under vacuum. THF (Sigma Aldrich, 99.9%, inhibitor‐free) underwent a three‐step purification/drying procedure, first with molecular sieves for five days, one day reflux with Na/K alloy, and fractional distillation. All electrolytes were prepared in an Ar‐filled glovebox (< 1 ppm O_2_, < 1 ppm H_2_O). Ionic liquid‐based Al electrolyte was prepared by gradual addition of AlCl_3_ to EMIMCl (in molar ratios 1.5:1) with continuous stirring. Amide‐based electrolytes were prepared in the same manner by slow addition of amides to AlCl_3_ (in molar ratios 1.5:1). After the addition, Al foil was added to all the electrolytes, and mixing was continued overnight. Clear solutions were obtained and used as an electrolyte.

All electrochemical experiments were performed in two‐electrode pouch cells, with two molybdenum current collectors (0.025 mm, 99.95%). One glassy fiber GF/A (20 mm) was used as a separator, and it was soaked in 100 μL of appropriate electrolyte. Al foil (0.1 mm, 99.99%) was washed with acetone, cut into 16 mm discs, dried at 80 °C overnight, and used as an anode.

Electrochemical measurements were performed in different voltage windows to estimate cycling stability. To account for the capacitive contribution of carbon black in electrode composition, tests with electrodes containing carbon black and binder in a weight ratio of 3:1 were performed under the same conditions as for electrode containing active material (same electrode preparation procedure as described above). The capacitive contribution was subtracted according to the previously published procedure.^[^ ^20^, ^29^ ^]^ Cycling was performed at different current densities, spanning from 50 mA g^−1^ to 1 A g^−1^.

##### Postmortem Chemical Analyses

Prior to *postmortem* chemical analyses of COF‐containing organic electrodes, electrodes were subjected to electrochemical pretreatment (cycling in the voltage range from 0.5 to 2.0 V, with the current density of 50 mA g^−1^), followed by disassembly inside the Ar‐filled glovebox and washing with dry THF (three times in 2 mL of THF, soaked each time for 10 min).

*Ex situ* electrodes were characterized using Bruker Alpha II spectrometer, on KBr pellets containing around 1% of the electrode diluted with KBr salt. The IR spectra were collected in transmittance mode with 48 scans at a resolution of 4 cm^−1^ in the range of 4000–500 cm^−1^.

SEM coupled with EDX (SEM/EDX) was performed on FE SEM Supra 35 VP Carl Zeiss equipped with Oxford Instrument Ultim Max 100 EDX detector. Analysis was performed at an accelerating voltage of 20 kV with the use of the SE2 detector. For sample transfer, a vacuum stage was used to avoid any exposure to ambient conditions. Data were collected at ten separate electrode spots and then averaged. Details of chemical and electrochemical analyses can be found in Supporting Information.

XRS measurements were performed at the ID20 beamline of the European Synchrotron Radiation Facility (ESRF).^[^ ^30^ ^]^ A liquid‐nitrogen cooled Si(111) premonochromator and a Si(311) postmonochromator were used to provide monochromatic incident X‐ray beam. The incoming photon beam was directed onto the sample surface at an angle of 7° relative to the horizontal plane. Samples were sealed in pouch cells to prevent air exposure. Inelastically scattered photons were analyzed by a large‐solid‐angle XRS spectrometer ^[^ ^1^ ^]^ consisting of an array of Johan‐type analyzer crystals arranged in three units in the vertical scattering plane. Each unit consists of 12 spherically bent Si(660) analyzers, each 100 mm in diameter, housed in a vacuum‐sealed movable module. The collection angles of the individual modules were fixed at 30°, 75°, and 121°, corresponding to momentum transfers q ≈2.5 Å^−1^, 6 Å^−1^, and 8.5 Å^−1^, respectively.

The overall experimental energy resolution was 0.72 eV, determined from the linewidth of the elastic scattering signal. For the O K‐edge measurements, we scanned the monochromator over the 10.197–10.257 keV range in 0.5 eV steps. After each scan, the sample was moved so that the beam irradiated a fresh spot on the surface, preventing radiation damage over prolonged exposure. We summed the spectra from five such points to obtain the final O K‐edge spectrum, with a total acquisition time of 50 min. For the Al L_2,3_‐edge, the monochromator was scanned over the 9.727–9.807 keV range in 0.5 eV steps. We summed the spectra from 5–8 such measurements at different locations on the sample surface for the final Al L_2,3_‐edge spectrum, leading to an overall acquisition time of 1–1.5 h.

To extract the final XRS spectra, we followed the reported procedure.^[^ ^31^ ^]^ For the O K‐edge spectrum, the signal from all three detector modules was summed. Because of the highest signal/background ratio, only the signal from the module positioned at 120° was used for the Al L_2,3_‐edge. The spectrum recorded at high momentum transfer contains dipole‐forbidden transitions, which enhance sensitivity to the coordination of Al ions.

## Conflict of Interest

The authors declare no conflict of interest.

## Supporting information

Supplementary Material

## Data Availability

The data that support the findings of this study are available from the corresponding author upon reasonable request.
