# Supplementary material for: Toward Practical Aluminum Organic Batteries Featuring Covalent Organic Framework
Source: ChemSusChem. 2025 Aug 5;18(19):e202500965. doi: 10.1002/cssc.202500965 (PMC12487733; doi:10.1002/cssc.202500965)
Supplement: Supplementary file 1 — Supplementary Material [file CSSC-18-e202500965-s001.pdf]

# Supporting Information for:

## Towards Practical Aluminum Organic Batteries Featuring Covalent Organic Framework

Olivera Lužanin,[1]* Raquel Dantas,[2] Ava Rajh,[3],[4] Urban Košir,[1],[5] Robert Dominko,[1],[5],[6] Klemen Bučar,[3],[4] Matjaž Kavčič,[3],[4] Manuel Souto,[2],[7]* Jan Bitenc[1]*

[1] Department of Materials Chemistry, National Institute of Chemistry, Ljubljana, 1001, Slovenia

[2] Department of Chemistry, CICECO-Aveiro Institute of Materials, University of Aveiro, Aveiro, 3810-393, Portugal

[3] Department of Low and Medium Energy Physics, Jožef Stefan Institute, Ljubljana, 1000, Slovenia

[4] Faculty of Mathematics and Physics, University of Ljubljana, Ljubljana, 1000, Slovenia

[5] Faculty of Chemistry and Chemical Technology, University of Ljubljana, Ljubljana, 1001, Slovenia

[6] ALISTORE – European Research Institute, CNRS FR 3104, Hub de l'Energie, Amiens, 80039, France

[7] CiQUS, Center for Research in Biological Chemistry and Molecular Materials, Department of Physical Chemistry, University of Santiago de Compostela, Santiago de Compostela, 15782, Spain

**Corresponding authors:** olivera.luzanin@ki.si, manuel.souto.salom@usc.es, and jan.bitenc@ki.si

## 1 Synthesis and Characterization of DAAQ-TFP-COF

All reagents for the synthesis of **DAAQ-TFP-COF** were of high purity grade and were purchased from Sigma Aldrich Co (diaminoanthraquinone) or TCI (1,3,5-triflormylphloroglucinol).

**Synthesis of DAAQ-TFP-COF** was carried out according to a reported procedure with minor modifications.[1] First, 1,3,5-triflormylphloroglucinol (TFP) (40.0 mg, 0.190 mmol), 2,6-diaminoanthraquinone (DAAQ) (68.0 mg, 0.285 mmol), dimethylacetamide (1.8 mL), and mesitylene (0.6 mL) were added into a Pyrex 8-mL vial. The suspension was sonicated at room temperature for five minutes. Then, 6 M acetic acid (100 μL) was added to the vial, and the suspension was sonicated again for a few minutes. The vial was then sealed and heated at 120 °C for three days. After cooling to room temperature, the reaction mixture was filtered and the precipitate was thoroughly washed with *N, N*-dimethylformamide (DMF), and acetone. The material was dried at 120 °C overnight, resulting in a dark red powder (85 mg, 86% yield).

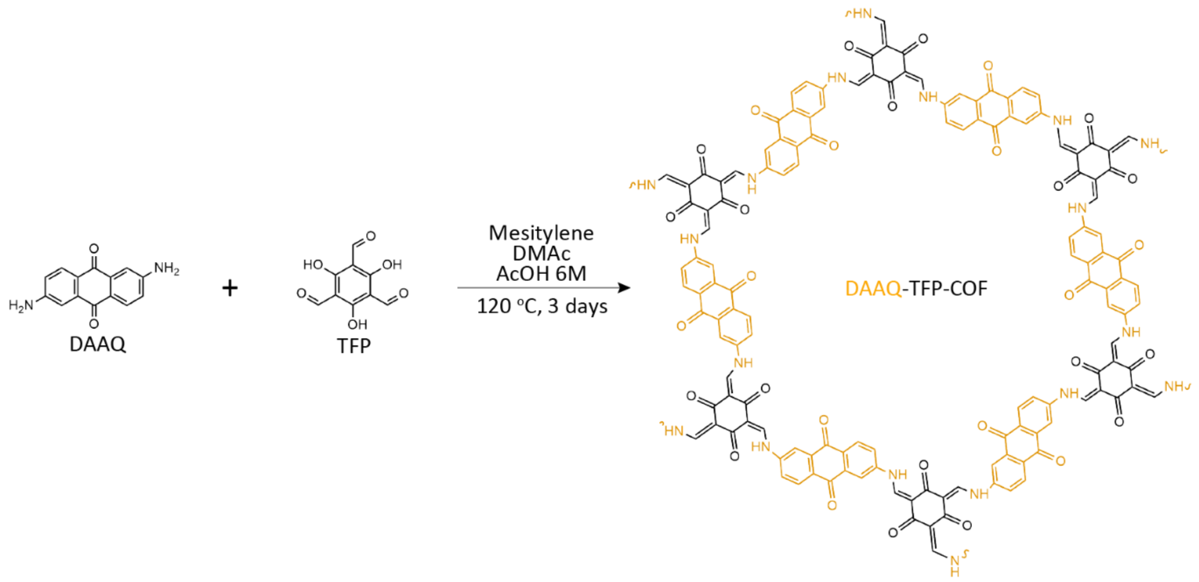


**Figure S1.** Scheme of DAAQ-TFP-COF synthesis.

**Powder X-ray diffraction (PXRD)** data were collected at 25 °C using a Rigaku Miniflex-600 diffractometer equipped with Cu Kα radiation (λ = 1.5406 Å). The measurements were conducted over a 2θ range of 2-35°, with a step size of 0.01° and a scanning rate of 0.4° $min^{-1}$. The sample was mounted on Si (511)-oriented single crystal substrate. The simulated PXRD pattern was generated from a previously published CIF file.[1] Pawley refinement was carried out using the X'Pert HighScore software package. A satisfactory Pawley refinement of DAAQ-TFP-COF was achieved, yielding a weighted R-factor (Rwp) of 1.97% and a Goodness of Fit (GOF) of 2.00.

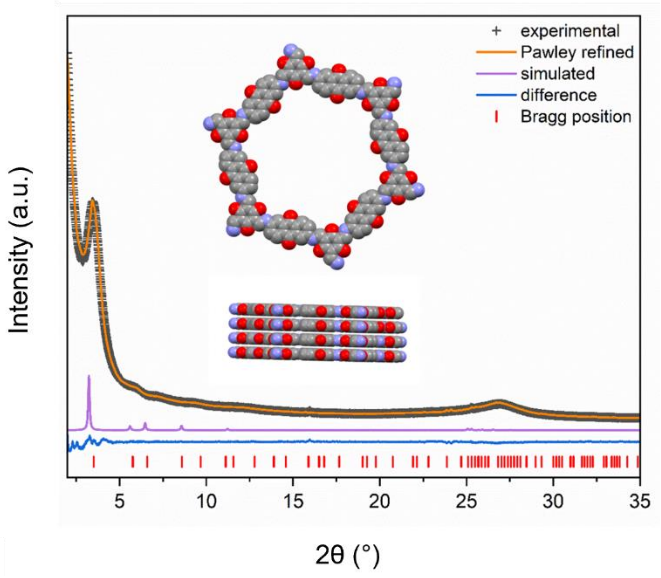


**Figure S2.** Experimental (black), simulated (purple), and Pawley refines (superimposed orange line) PXRD patterns of DAAQ-TFP-COF in conjugation with the difference plot (blue) and the Bragg position representation (red) using the computational model with AA stacking mode. The inset shows top and lateral views of the COF model.

**Infrared spectroscopy (IR)** spectra were recorded on Bruker Tensor 27 spectrometer coupled with a Specac Golden Gate Diamond attenuated total reflectance (ATR) accessory. Measurements were recorded in the range between 4000 and 350 $cm^{-1}$ with a resolution of 4 $cm^{-1}$ and 256 scans. IR spectroscopy confirms the formation of β-ketoenamine linkages in DAAQ-TFP-COF since the spectrum shows the emergence of a new C-N stretch at approximately 1250 $cm^{-1}$, as well as the disappearance of the N-H stretch characteristic of the DAAQ precursor. Furthermore, the spectrum also lacks OH stretches, indicating the absence of imine tautomers. Additionally, the C=O stretch originally present in the TFP precursor at 1658 $cm^{-1}$ shifts to approximately 1615 $cm^{-1}$, consistent with the conjugated C=O vibration in the keto form.[1]

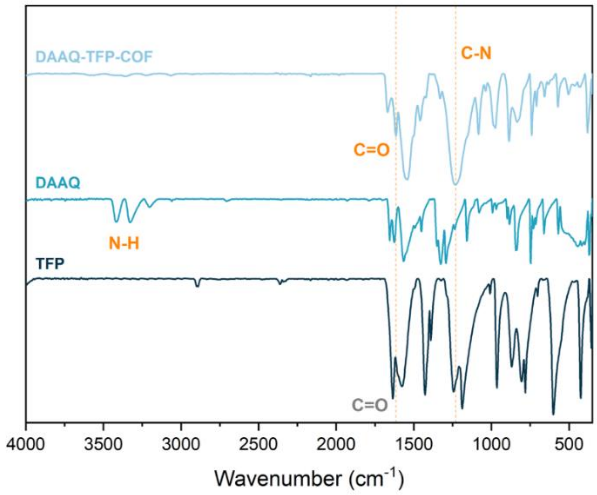


**Figure S3.** IR spectra of starting reagents (TFP and DAAQ) and the obtained COF (DAAQ-TFP-COF).

**Solid-state $^{13}C$ cross-polarization magic angle spinning nuclear magnetic resonance (CPMAS NMR)** was performed on a Bruker Avance III 400 MHz spectrometer equipped with a 4 mm NMR probe. $^{13}C$ CPMAS solid-state NMR spectroscopy indicated the formation of the β-ketoenamine linkage, as the spectra exhibit a resonance at 143 ppm that is assigned to the enamine carbon (=CNH).[1]

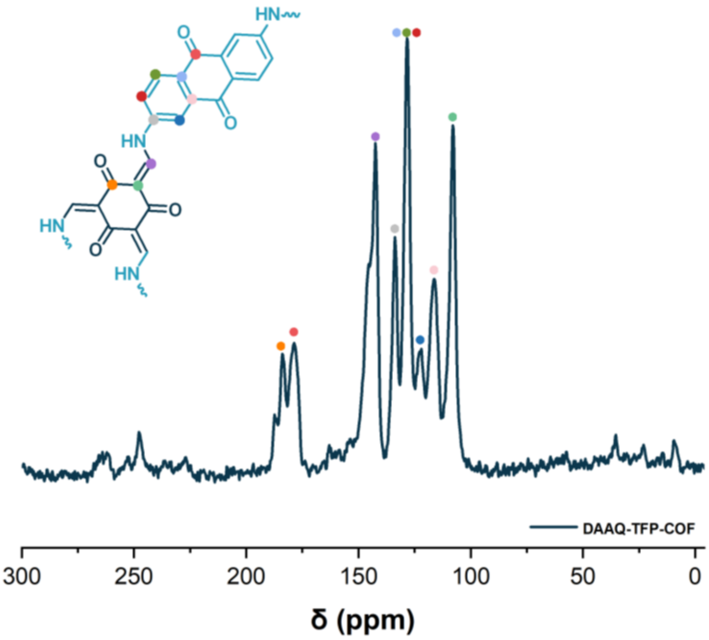


**Figure S4**. Solid-state CPMAS $^{13}C$ NMR of DAAQ-TFP-COF.

**Thermogravimetric analysis (TGA)** was carried out with a Shimadzu TGA 50 equipment in the 25-600 °C temperature range under a 5 °C min$^{-1}$ scan rate and an $N_2$ flow of 50 mL min$^{-1}$.

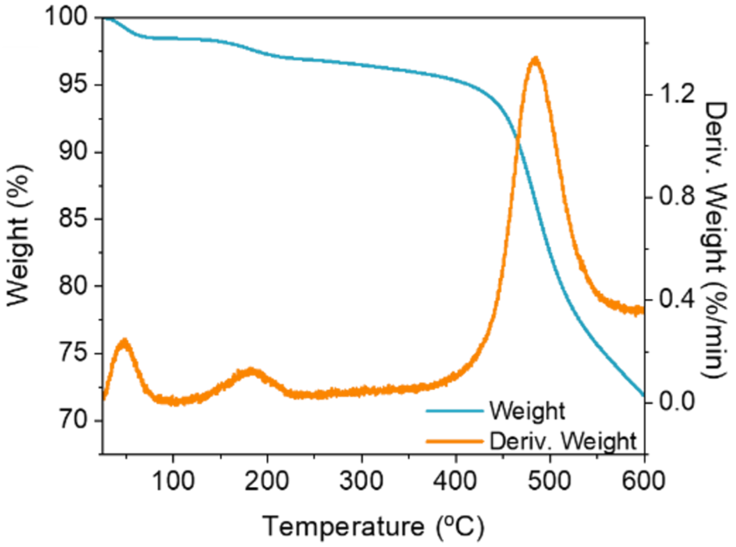


**Figure S5.** TGA profile and DFT curve of DAAQ-TFP-COF.

$N_2$ isotherm was collected at 77 K using a 3FLEX™ (Micrometrics). Before measurements, the sample was outgassed at 393 K and $10^{-6}$ Torr overnight using Smart VacPrep (Micrometrics) equipment. BET surface value was calculated from the $N_2$ isotherm using BETSI.[2] Pore size distributions were obtained using the nonlocal density functional theory (NLDFT) method.

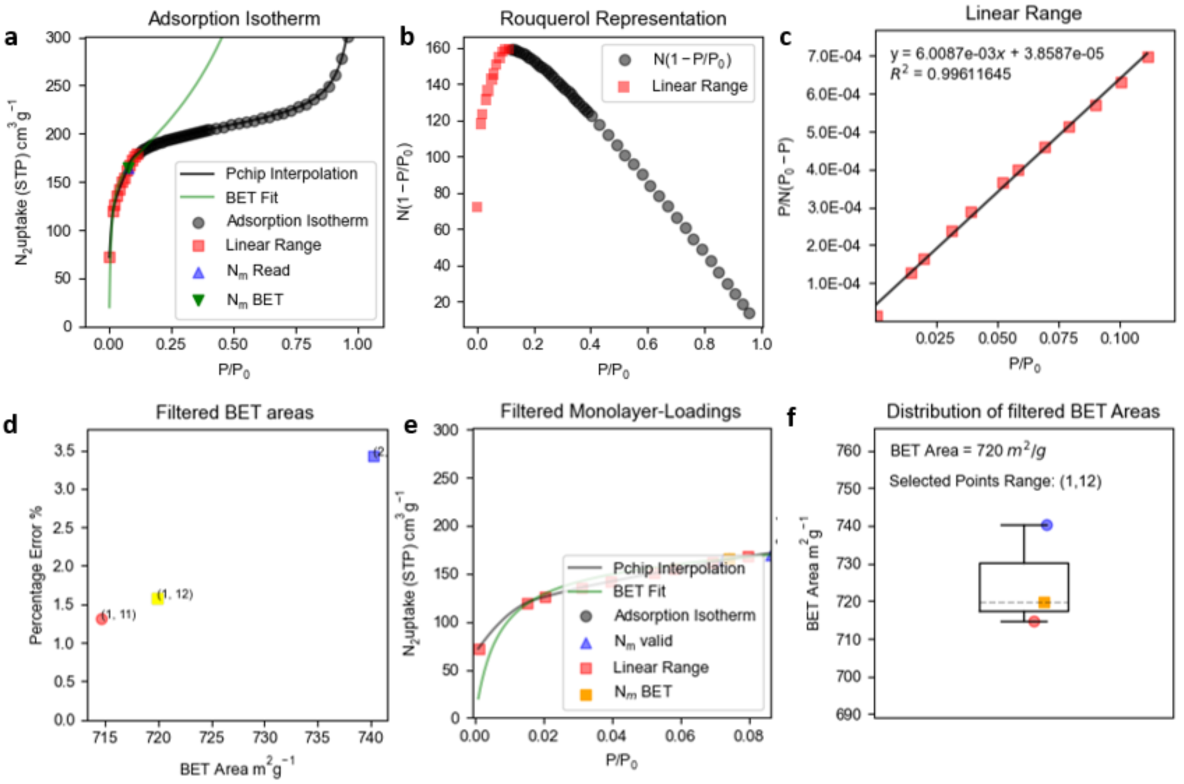


**Figure S6.** BETSI regression diagnostics for DAAQ-TFP-COF. The BET surface area was calculated to be 720 m$^2$ g$^{-1}$.

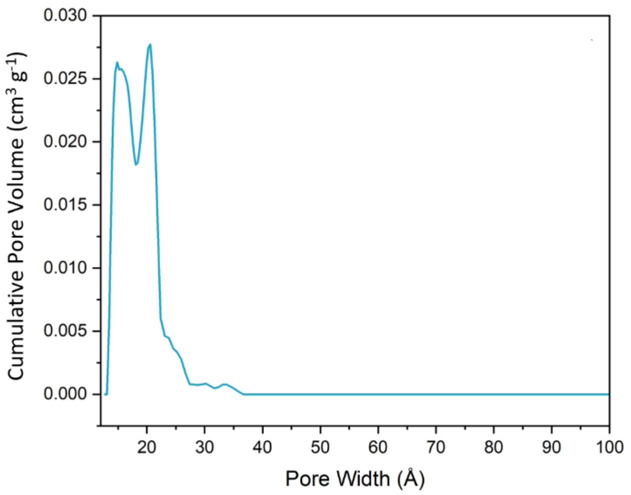


**Figure S7.** Pore size distribution curve of DAAQ-TFP-COF.

**Elemental analysis** (CNSH) was performed using a Thermo FlashSmart Elemental Analyzer 1112 (Thermo Fisher).

**Table S1.** Elemental analysis of DAAQ-TFP-COF compared with calculated values for $C_{10}H_5N_1O_2$ (empirical formula of DAAQ-TFP-COF).

| | C (wt.%) | H (wt.%) | N (wt.%) |
|---|---|---|---|
| **Theoretical** | 70.18 | 2.94 | 8.18 |
| **Measured** | 65.49 | 4.54 | 7.33 |

**Scanning electron microscopy (SEM)** was performed on a high-resolution Hitachi SU-70 with an accelerating voltage of 15 kV.

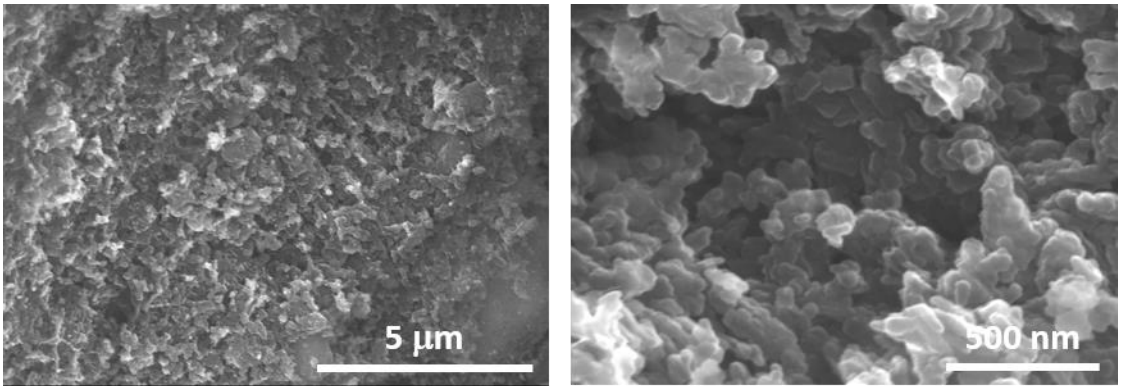


**Figure S8.** SEM images of DAAQ-TFP-COF powder.

**Theoretical capacity calculation:** The theoretical capacity of DAAQ-TFP-COF was calculated using equation (1), where *F* represents Faraday's constant of 96485 C $mol^{-1}$ = 26 801 mAh $mol^{-1}$; Mw is the molar mass of the active material in g $mol^{-1}$; *n* corresponds to the number of electrons expected in the redox reaction of the active material.

$$C_{th} = \frac{n\,F}{M_w} \quad (1)$$

The molecular weight of the DAAQ-TFP-COF unit cell is equal to 1062 g $mol^{-1}$ and considering the total of 6 active sites (three anthraquinones, each containing two C=O active sites in turn corresponding to 6 electrons per unit cell), consequently, the theoretical capacity is calculated as 151 mAh $g^{-1}$.[1]

**Table S2.** Overview of recently reported organic compounds with Al charge carriers, with cathode compositions, achieved capacities, and corresponding structures. Below the table are displayed molecular structures of organic compounds with the abbreviations used in the table.

| Compound | Electrolyte | Cathode composition (AM : CB : Binder) | Voltage window (V) | $C_{meas}$ (mAh $g^{-1}$) (current density) | Coordination species | Technique(s) | Ref. |
|---|---|---|---|---|---|---|---|
| NiTPP | 1.3 EMIMCl | 6:3:1, PVdF | 0.1-2.3 | 177.1 (50 mA $g^{-1}$) | $AlCl_2^+$ | XPS, EXAFS, XAS | [3] |
| 2D-NT-COF30 | 1.3 EMIMCl | 7:2:1 (Super P, PVdF + 30 wt.% CNTs) | 0.5-2.3 | 132 (100 mA $g^{-1}$) | $AlCl_2^+$ | XPS, EDX, $^{27}Al$ NMR | [4] |
| $H_2TPP$ | 1.3 EMIMCl | 6:3:1, PVdF | 0.2-2.2 | 101 (100 mA $g^{-1}$) | $AlCl_2^+$ | $^{27}Al$ NMR, TOF-SIMS | [5] |
| PHATN | 1.5 $AlCl_3$-[BMIm]Cl | NA | 0.2-1.2 | 143 (50 mA $g^{-1}$) | $AlCl_2^+$ | EDX | [6] |
| SPTCDA | EMIMCl | NA | 0.1-2.3 | 252 (1 A $g^{-1}$) | $AlCl_2^+$ | DFT | [7] |
| DAAQ-TP/CNT | 1.3 EMIMCl | 6:3:1 (Ketjenblack, CMC) | 0.3-2.2 | 300 (0.2 A $g^{-1}$) | $AlCl^{2+}$ | TOF-SIMS, EDX | [8] |
| BDTP | $AlCl_3$:TMAHCl=1.8 | 7:2:1 (sodium alginate, Super P) | 0-1.8 | 137 (20 mA $g^{-1}$) | $AlCl_2^+$ | XPS, EDX, TOF-SIMS | [9] |
| AQ | 1.5 EMIMCl | 8:1:1 (Acetylene black, PTFE) | 0.1-2.1 | 215 (100 mA $g^{-1}$) | $Al^{3+}$ | EDX, ICP, XPS | [10] |
| BDTO/MXene | 1.3 EMIMCl | 5:3:2 ((AM:Mxene 1:2), Acetylene black, PVdF) | 0.1-2.4 | 301 (500 mA $g^{-1}$) | $Al^{3+}$ | XPS, EDX | [11] |
| PAQS/CNT | 1.5 EMIMCl | 6:3:1 (Printex XE2, PTFE) | 0.4-1.8 | 190 (0.11 A $g^{-1}$) | $AlCl^{2+}$ | EDX, XPS | [12] |
| PQ | 1.3 EMIMCl | 3:5:2 (Denka black, PVdF) | 0.7-1.75 | 94 (0.2 A $g^{-1}$) | $AlCl_2^+$ | EDX, TOF-SIMS | [13] |
| TPBQ | 1.3 EMIMCl | 5:1:3:1 (graphene, Ketjen black, PTFE) | 0.5-2.3 | 177 (20 mA $g^{-1}$) | $AlCl_2^+$ | XPS, EDX | [14] |
| PYTQ/CNT | $AlCl_3$:urea=1.3 | 6:3:1 (Ketjen black, CMC) | 0.1-2.1 | 208 (0.2 A $g^{-1}$) | $AlCl_2(urea)_2^+$ | XPS, EDX | [15] |
| IEP-27-SR | 1.5 EMIMCl | 1:1 (SWCNTs, rGO) | 0.2-1.6 | 116 (97 mA $g^{-1}$) | $AlCl_2^+$ | EDX | [16] |

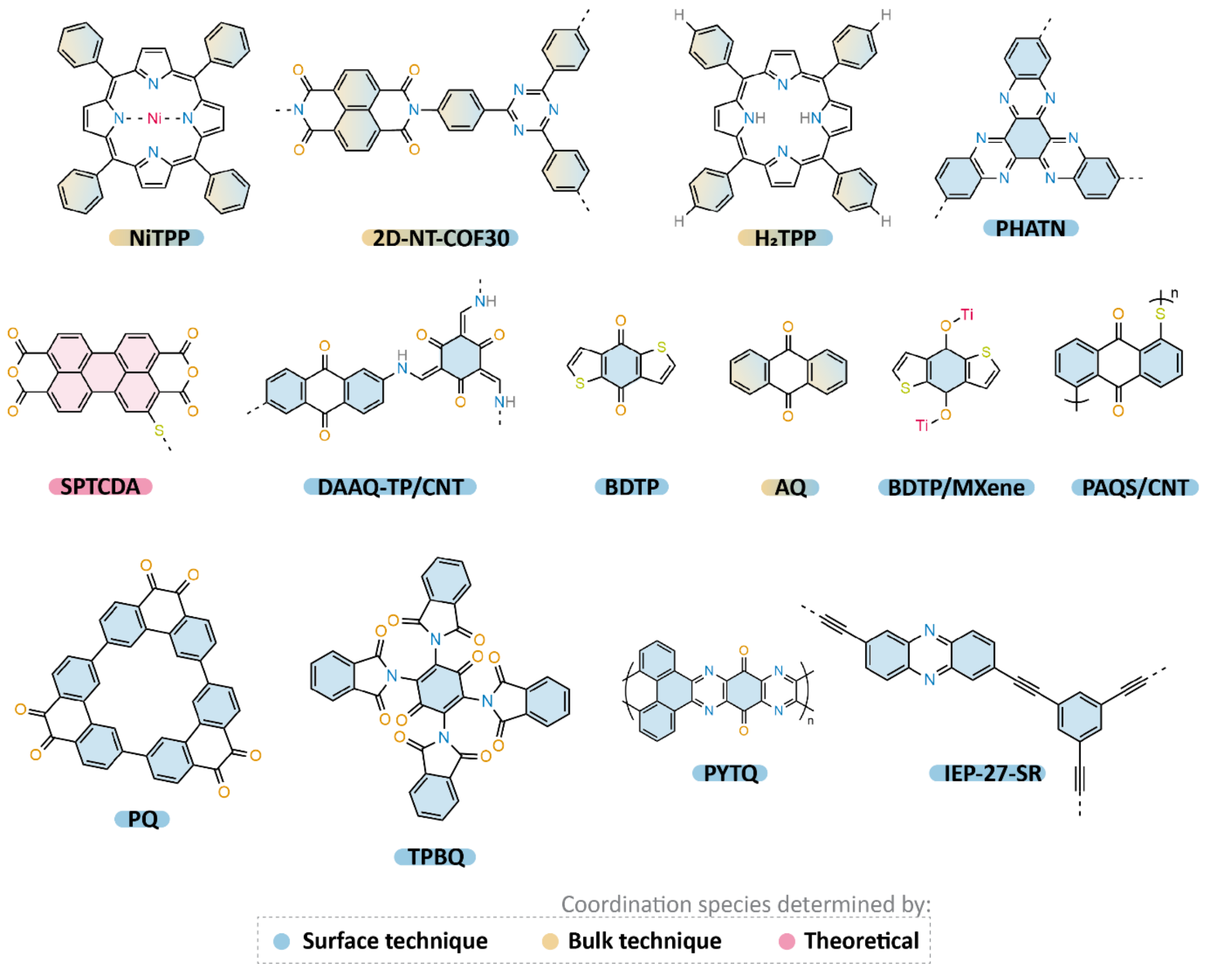
NiTPP
2D-NT-COF30
H₂TPP
PHATN
SPTCDA
DAAQ-TP/CNT
BDTP
AQ
BDTP/MXene
PAQS/CNT
PQ
TPBQ
PYTQ
IEP-27-SR
Coordination species determined by:
Surface technique
Bulk technique
Theoretical

## 2 Ionic Liquid Electrolyte Performance

Electrolyte stability was assessed in a two-electrode pouch cell with molybdenum current collectors, one GF/A separator soaked with 100 µl of 1.5EA electrolyte, and a molybdenum disc as the working electrode. The applied sweep rate was 0.1 mV $s^{-1}$.

Aluminum plating/stripping experiment was performed in the same setup. Plating on molybdenum was performed for 60 minutes at 0.1 mA $cm^{-2}$ current density, followed by Al stripping until a cutoff voltage of 1.5 V. The process was repeated for 100 cycles.

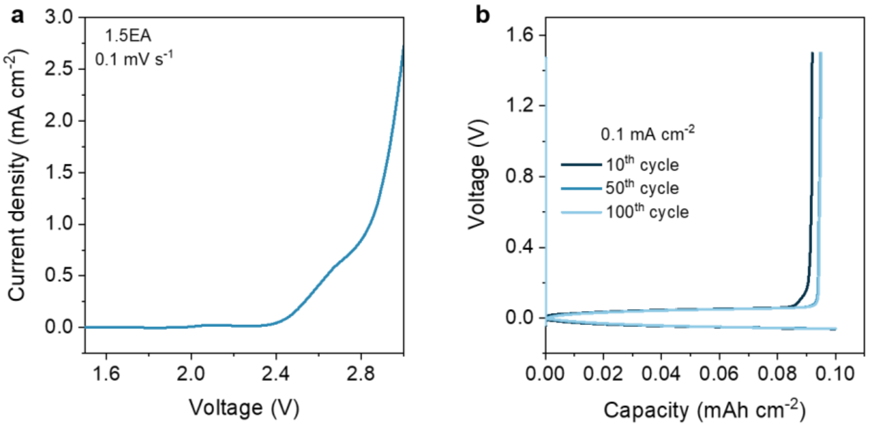


**Figure S9.** 1.5EA electrolytes assessment. a) Linear sweep voltammetry with molybdenum foil as WE, measured at a sweep rate of 0.1 mV $s^{-1}$. b) Selected voltage profiles of Al plating/stripping.

Galvanostatic cycling of carbon black:PTFE (3:1, wt%) electrodes was performed in two-electrode pouch cells, with molybdenum current collectors. Cycling was performed at a current density of 50 mA $g^{-1}$ in three different voltage windows (0.3-1.8 V, 0.3-2.0 V, 0.3-2.2 V).

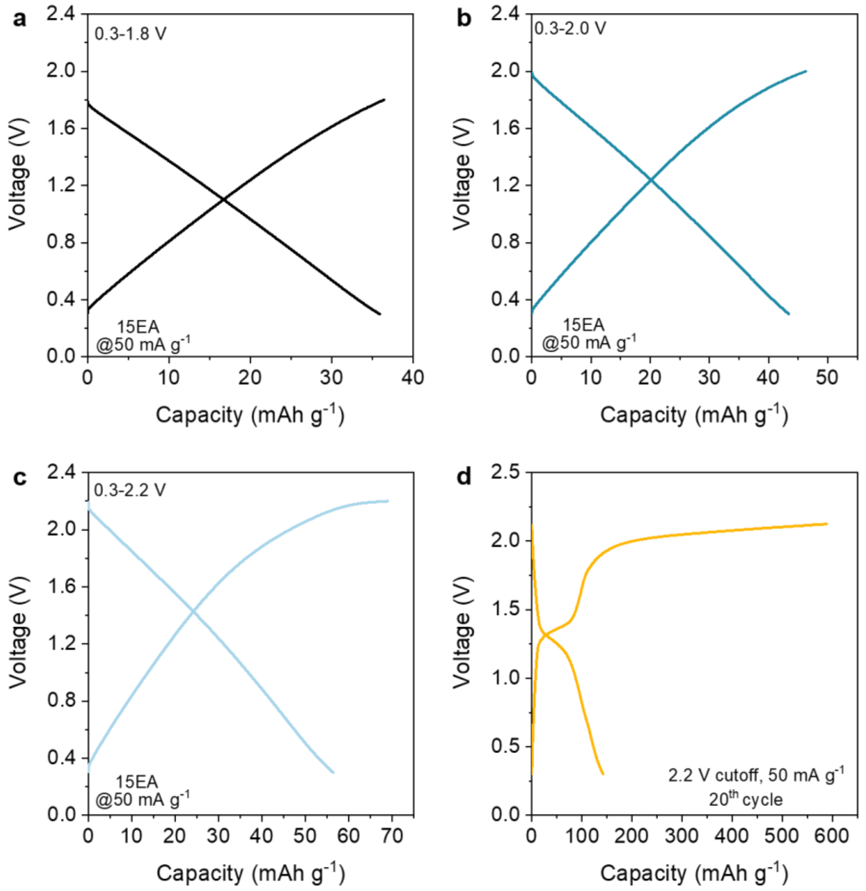


**Figure S10.** Printex:PTFE (3:1 wt.%) cycling in 15EA at 50 mA $g^{-1}$ ($5^{th}$ cycle) in different voltage windows spanning a) 0.3-1.8 V, b) 0.3-2.0 V, and c) 0.3-2.2 V, with d) $20^{th}$ charge/discharge cycle of DAAQ-TFP-COF cathode obtained in 1.5EA with upper cutoff of 2.2 V.

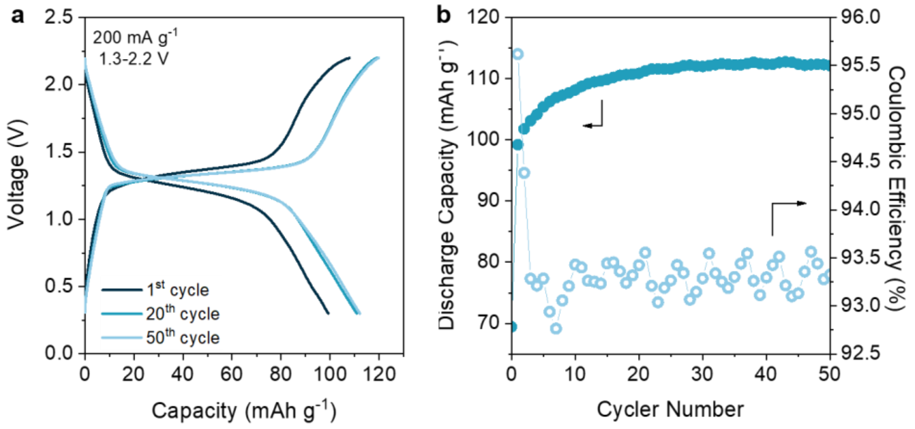


**Figure S11.** Electrochemical performance of DAAQ-TFP-COF cathode in 1.5EA in a voltage span from 0.3 to 2.2 V at a current density of 200 mA $g^{-1}$. a) Selected galvanostatic charge/discharge voltage profiles. b) Discharge capacity and Coulombic efficiency evolution over 200 cycles.

## 3 Capacitance Contribution Subtraction

Considering that the electrodes employed in the experiments outlined in this manuscript contain 30 wt% of high surface area carbon black, in order to obtain real information about material utilization, the capacitive contribution stemming from carbon black needs to be subtracted. In order to perform such subtraction, we have first prepared electrodes containing just Printex XE2 carbon black and PTFE binder in a weight ratio of 3:1, mimicking the ratio between CB and binder in organic electrodes. Electrodes were prepared following the procedure described in the Materials and Methods section.

Electrochemical performance assessment was performed in two-electrode pouch cells, with molybdenum current collectors and Al metal foil as counter/reference electrode. Current densities were the same as for cycling of the electrode containing active material. The capacitive contribution was subtracted following the published procedure, using the formula:[17]

$$C_{AM} = C_{measured} - \frac{m_{CB}}{m_{AM}} C_{CB} \qquad (2)$$

where $C_{AM}$, $C_{measured}$, and $C_{CB}$ represent capacities of active material, measured capacity on the full electrode, and capacity of carbon black, and a correction factor consisting of the mass percentage of carbon black divided by the mass percentage of active material. In our case, the electrode contained active material and carbon black in a weight ratio of 6:3, meaning that the correction factor has a value of 0.5.

**Table S3.** Measured capacities of electrodes containing active material, carbon black, and PTFE binder in weight ratio 6:3:1; measured capacities of electrodes containing carbon black and PTFE binder in weight ratio 3:1; capacity of active material, obtained using the measured capacities and formula (1).

| | Current density (mA $g^{-1}$) | | | | |
|---|---|---|---|---|---|
| | 50 | 100 | 200 | 500 | 1000 |
| $C_{measured}$ (mAh $g^{-1}$) | 113.9 | 108.9 | 102.5 | 90.1 | 74.5 |
| $C_{CB}$ (mAh $g^{-1}$) | 28.2 | 27.6 | 26.8 | 25.4 | 22.9 |
| $C_{AM}$ (mAh $g^{-1}$) | 99.8 | 95.1 | 89.1 | 77.4 | 63.1 |

## 4 DAAQ-TFP-COF Activation

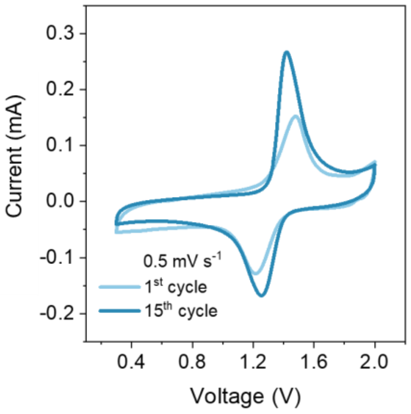


**Figure S12.** Cyclic voltammograms recorded at scan rate 0.5 mV $s^{-1}$, showing first and tenth CV curve, with peaks at 1.2 V and 1.4 V increasing in intensity, indicative of DAAQ-TFP-COF activation.

Electrochemically assisted swelling was performed following the published procedure.[18] First, a half-cell containing DAAQ-TFP-COF cathode and Li metal counter electrode was assembled, using one GF/A separator soaked in 60 µl of 1 M LiTFSI in DOL/DME (1:1, vol%). The cathode was discharged down to 1.5 V and then charged back to 3.5 V with a current of 10 mA $g^{-1}$. The electrode was then harvested from a cell, thoroughly washed with DME, and dried overnight. After drying, the electrode was paired with an Al metal anode and 1.5EA electrolyte, according to the procedure described in the Materials and Methods Section.

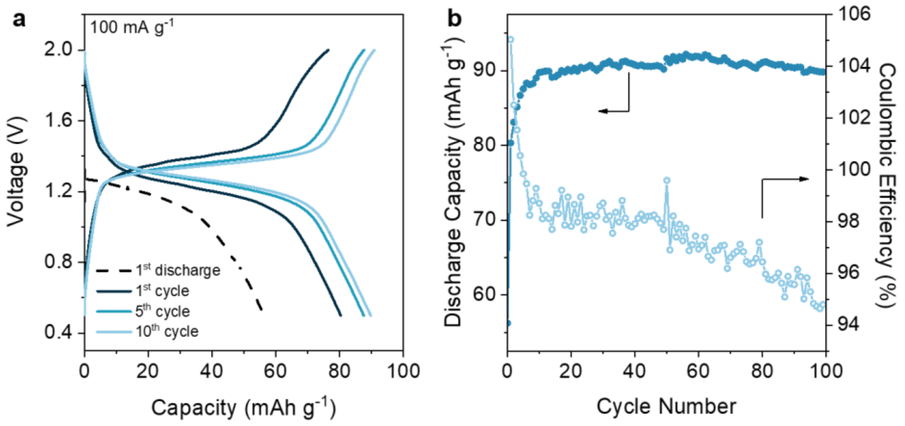


**Figure S13.** Electrochemically assisted swelling. a) Selected galvanostatic charge/discharge profiles obtained at 100 mA $g^{-1}$. b) Discharge capacity evolution over 100 cycles with corresponding Coulombic efficiency.

# 5 *Ex Situ* Analyses

## ***Infrared spectroscopy***

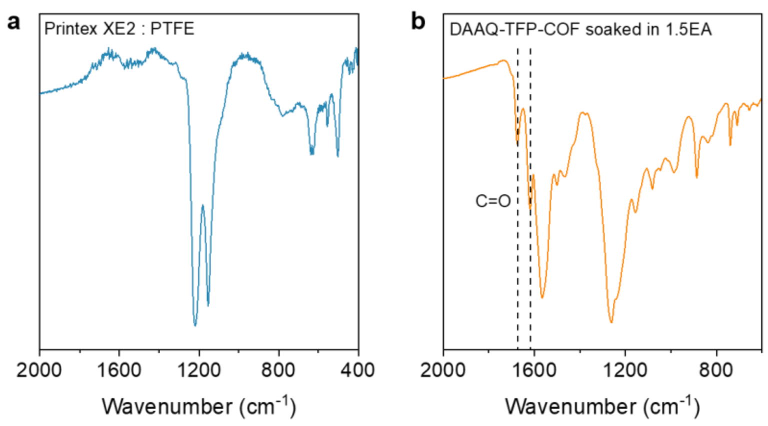


**Figure S14.** IR spectra of a) Printex XE2:PTFE (3:1 wt.%) electrode, showing the characteristic peaks of PTFE at 1219 and 1155 $cm^{-1}$ but no interference with the C=O region of interest; b) soaked DAAQ-TFP-COF electrode showing no changes upon 24 h soaked in 1.5EA.

## ***EDX Analyses***

Measurements were performed on FE SEM Supra 35 VP Carl Zeiss equipped with Oxford Instruments Ultim Max 100 EDX detector. Atomic percentages shown in the table below were obtained at an accelerating voltage of 20 kV, with the use of an SE2 detector. For each sample (pristine, soaked, charged, and discharged electrodes as well as electrodes containing only carbon black) EDX spectra were obtained in 10 different spots, and values shown in Table S3 are averaged.

**Table S4.** Average atomic percentages of C, O, F, Al, Cl, and calculated Cl:Al ratios obtained on pristine, soaked, discharged, charged, and carbon black (CB) electrodes. Electrodes were prepared for the analysis following the procedure described above. Values were measured on ten independent spots and averaged.

| | C | O | F | Al | Cl | Cl:Al |
|---|---|---|---|---|---|---|
| **Pristine** | 86.7 | 8.4 | 4.8 | 0 | 0 | - |
| **Soaked** | 84.5 | 8.3 | 4.3 | 1.2 | 1.7 | 1.4 |
| **Discharged** | 79.6 | 9.5 | 3.8 | 2.6 | 4.5 | 1.7 |
| **Charged** | 82.9 | 8.9 | 4.3 | 1.5 | 2.4 | 1.6 |
| **CB electrode** | 83.0 | 2.0 | 12.9 | 0.6 | 1.5 | 2.5 |

***XRS Analyses***

Standards for XRS measurements were prepared according to the previously reported procedure inside the Ar-filled glovebox ($O_2$ and $H_2O$ levels below 1 ppm).[19] Anthraquinone (Fluka, 99%; 208 mg, 1 mmol) was dissolved in DMSO (Sigma-Aldrich, 99,9%; 10 mL). Sodium borohydride (40 mg, 1 mmol) was added to the solution and the reaction mixture was stirred for 6 h at room temperature. Afterwards, it was poured into 1 M HCl (50 mL) to obtain precipitate, which was then filtered, washed several times with water, and dried under vacuum at 50 °C overnight. Fluorescent yellow 9,10-dihydroxy anthracene ($H_2AQ$) was obtained. Al salts of AQ-2($AlCl_2$) and AQ-AlCl were prepared by dissolving $H_2AQ$ (0.25 mmol) in 2 mL of THF solvent and reacting them by slow addition of $EtAlCl_2$ (0.50 mmol, 1.0 M in hexanes, Sigma Aldrich) or $Me_2AlCl$ (0.25 mmol, 1.0 M in hexanes Sigma Aldrich), respectively. Afterwards the reaction mixtures were mixed overnight and finally dried to remove THF and hexanes solvents to obtain model salts of AQ-2($AlCl_2$) and AQ-AlCl.

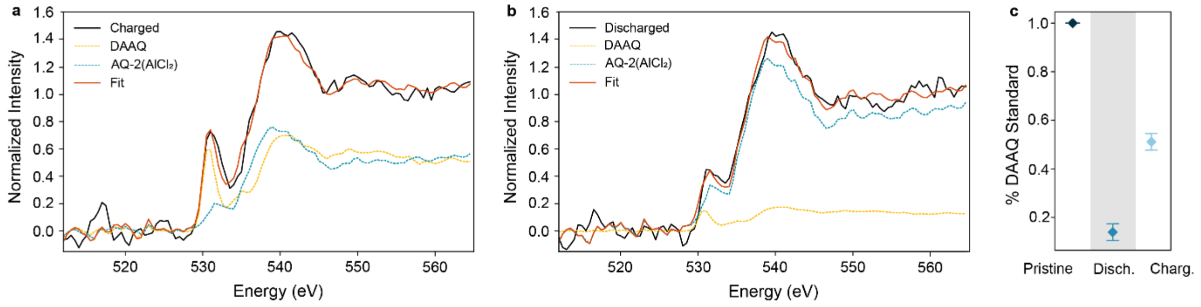


**Figure S15.** Linear combination fit (LCF) of DAAQ-TFP-COF electrodes using pristine DAAQ-TFP-COF (denoted as DAAQ) and AQ-2($AlCl_2$) standard to fit the spectrum of a) charged DAAQ-TFP-COF and b) discharged DAAQ-TFP-COF. c) Comparison of the relative amount of DAAQ carbonyl bond in pristine, charged, and discharged electrodes using LCF analysis of measured spectra.

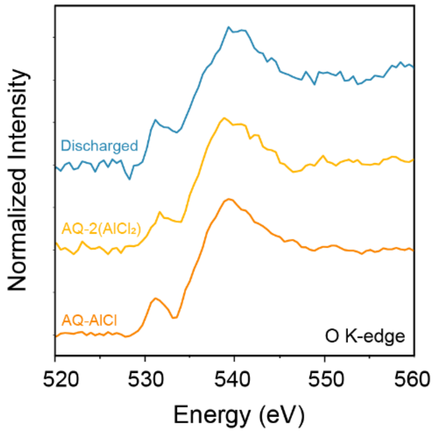


**Figure S16.** Oxygen K-edge comparison of discharged electrode spectra and two model compounds – AQ-2($AlCl_2$) and AQ-AlCl.

## 6 Energy Density Estimation

Theoretical anode capacity is strongly dependent on the amount of $AlCl_3$ that acts as anolyte. Different scenarios exist, depending on which species is formed, namely $Al^{3+}$, $AlCl_4^-$, $AlCl^{2+}$, and $AlCl_2^+$.[20] Considering the $AlCl_2^+$ identified as the main species present within the electrode, we will consider the particular case in which:

$$\mathrm{Al} + 2\mathrm{AlCl_3} \rightarrow 3\mathrm{AlCl_2^+} + 3\mathrm{e^-} \quad (3)$$

Then, we can calculate the capacity of the anode, following the formula in which *z* represents the number of exchanged electrons, and *r* is the ratio between $AlCl_3$ and EMIMCl. To maximize the anode capacity the r ratio is considered to be 2, the highest molar ratio that still forms applicable liquid electrolyte.

$$\mathrm{C_A} = \frac{\mathrm{z \times F}}{2\frac{\mathrm{rM_{AlCl_3} + M_{EMIMCl}}}{\mathrm{r-1}} + \mathrm{M_{Al}}} = 94.2\ \mathrm{mAh\ g^{-1}} \quad (4)$$

The maximal capacity DAAQ-TFP-COF cathode achieved was 113.9 mAh $g^{-1}$, at 50 mA $g^{-1}$. Therefore, the cell capacity (employing Al and DAAQ-TFP-COF cathode) can be calculated as:

$$\mathrm{C_{cell}} = \frac{\mathrm{C_A \times C_c}}{\mathrm{C_A + C_c}} = 51.6\ \mathrm{mAh\ g^{-1}} \quad (5)$$

Finally, taking into account the average discharge voltage of 1.2 V, the cell energy density is:

$$\mathrm{W_{cell}} = \mathrm{C_{cell}} \times \mathrm{E} = 62\ \mathrm{Wh\ kg^{-1}} \quad (6)$$

In case of divalent $AlCl^{2+}$ being the predominant species, the reaction would change to:

$$2\mathrm{Al} + \mathrm{AlCl_3} \rightarrow 3\mathrm{AlCl^{2+}} + 6\mathrm{e^-} \quad (7)$$

And, concomitantly, the anode capacity would change, following the equation:

$$\mathrm{C_A} = \frac{\mathrm{z \times F}}{\frac{\mathrm{rM_{AlCl_3} + M_{EMIMCl}}}{\mathrm{r-1}} + 2\mathrm{M_{Al}}} = 344\ \mathrm{mAh\ g^{-1}} \quad (8)$$

Therefore, the $C_{cell}$ would increase to 85.6 mAh $g^{-1}$, and with an average voltage of 1.2 V, this would result in a specific energy density of 103 Wh $kg^{-1}$.

# 7 Amide-Based Electrolytes Performance

Electrolyte stability was assessed in two-electrode pouch cells with molybdenum current collectors, one GF/A separator soaked with 80 µL of 1.5EA electrolyte, and a molybdenum disc as the working electrode. The applied sweep rate was 0.1 mV $s^{-1}$.

Aluminum plating/stripping experiment was performed in the same setup. Plating was performed for 60 minutes at 0.1 mA $cm^{-2}$ current density, followed by Al stripping until a cutoff voltage of 1.5 V. The process was repeated for 100 cycles.

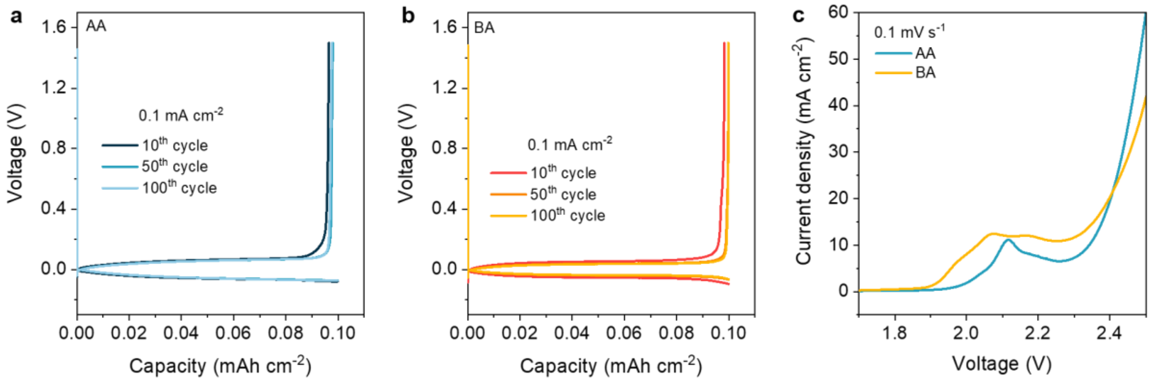


**Figure S17.** Amide-based electrolyte assessment. a) Selected voltage profiles of Al plating/stripping obtained in AA electrolyte at 0.1 mA $cm^{-2}$. b) Selected voltage profiles of Al plating/stripping obtained in BA electrolyte at 0.1 mA $cm^{-2}$. c) Linear sweep voltammetry with molybdenum foil as WE, measured at a sweep rate of 0.1 mV $s^{-1}$.

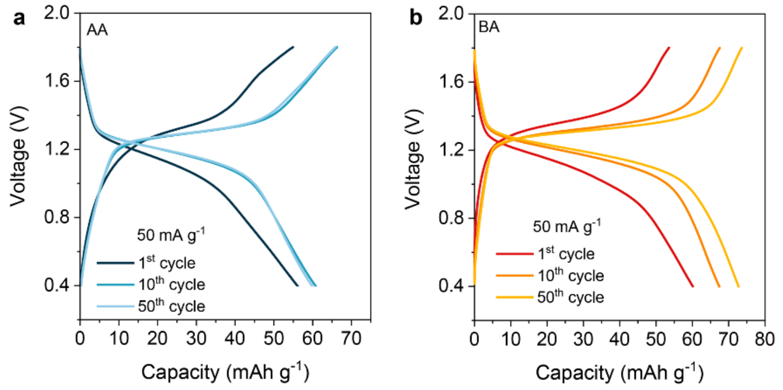


**Figure S18.** Galvanostatic charge/discharge voltage curve evolution with cycling at 50 mA $g^{-1}$ in a) acetamide (AA) electrolyte and b) butyramide (BA) electrolyte.

**Table S5.** Prices of the compounds used in the formulation of Al electrolytes, recalculated in €/100 g. The specified qualities of the chemicals are given in brackets, and the highest available purity for each compound with both providers was taken into account. Prices were obtained from https://www.sigmaaldrich.com/SI/en and https://www.fishersci.com/us/en/home.html on June 26, 2025. When prices were displayed in US dollars, an EUR:USD conversion rate of 1.17 was used.

| | **AA** (CAS: 60-35-5) | **BA** (CAS: 541-35-5) | **EA** (CAS: 65039-09-0) |
|---|---|---|---|
| **Sigma / Merck** | 15 (≥99.0 %) | 121 (≥98.0 %) | 479 (98 %) |
| **Fisher Scientific** | 12 (99%) | 80 (98%) | 1096 (98+%) |

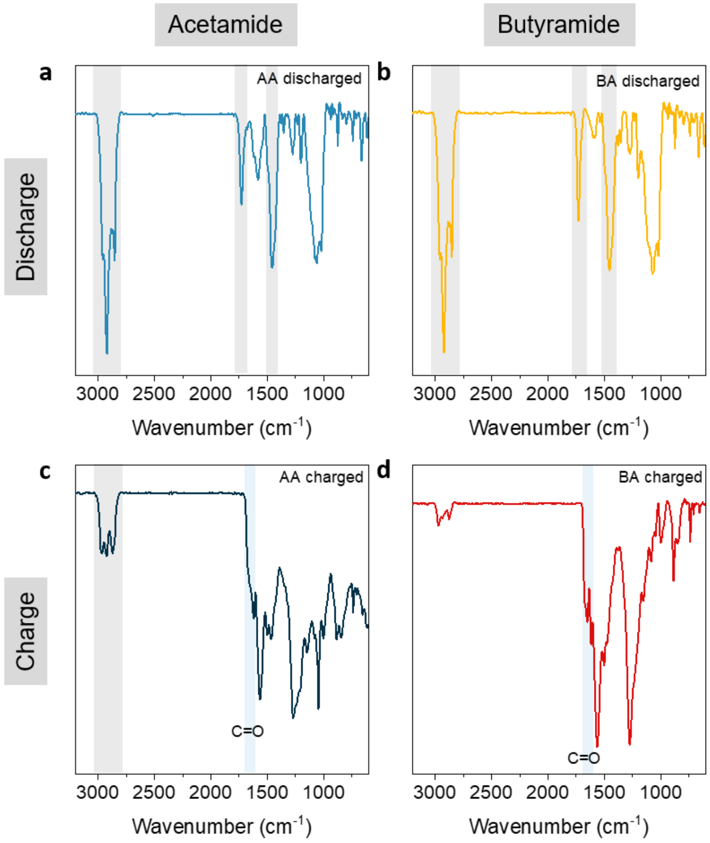


**Figure S19.** Infrared spectra of DAAQ-TFP-COF electrodes discharged down to 0.4 V in a) AA and b) BA electrolytes and charged to 1.8 V in c) AA and d) BA. Samples were washed and measured following the procedure provided in Materials and Methods Section.

**Table S6.** Average atomic percentages of C, O, F, Al, Cl, and calculated Cl:Al ratios obtained on soaked and electrodes discharged in amide-based electrolytes. Electrodes were prepared for the analysis following the procedure described above. Values were measured on ten independent spots and averaged, provided with relative error.

| | C | O | N | F | Al | Cl | Cl:Al |
|---|---|---|---|---|---|---|---|
| **Discharged AA** | 77.8 | 2.2 | 7.1 | 9.9 | 1.3 | 1.8 | 1.4 |
| **Discharged BA** | 75.3 | 1.9 | 8.0 | 12.2 | 1.1 | 1.4 | 1.3 |

## 8 References


[1] C. R. Deblase, K. E. Silberstein, T. T. Truong, H. D. Abruña, W. R. Dichtel, "β-Ketoenamine-Linked Covalent Organic Frameworks Capable of Pseudocapacitive Energy Storage"*J. Am. Chem. Soc.* **2013**, *135*, 16821–16824.

[2] J. W. M. Osterrieth et al., "How Reproducible are Surface Areas Calculated from the BET Equation?"*Adv. Mater.* **2022**, *34*, 2201502.

[3] S. Jiao, X. Han, X. Bu, Z. Huang, S. Li, W. Wang, M. Wang, Y. Liu, W. L. Song, "d-Orbital Induced Electronic Structure Reconfiguration toward Manipulating Electron Transfer Pathways of Metallo-Porphyrin for Enhanced $AlCl_2^+$ Storage" *Adv. Mater.* **2024**, *36*, 2409904.

[4] Y. Liu, Y. Lu, A. H. Khan, G. Wang, Y. Wang, A. Morag, Z. Wang, G. Chen, S. Huang, N. Chandrasekhar, D. Sabaghi, D. Li, P. Zhang, D. Ma, E. Brunner, M. Yu, X. Feng, "Redox-Bipolar Polyimide Two-Dimensional Covalent Organic Framework Cathodes for Durable Aluminum Batteries" *Angew. Chemie Int. Ed.* **2023**, *62*, e202306091.

[5] X. Han, S. Li, W. L. Song, N. Chen, H. Chen, S. Huang, S. Jiao, "Stable High-Capacity Organic Aluminum-Porphyrin Batteries" *Adv. Energy Mater.* **2021**, *11*, 2101446.

[6] M. Mao, C. Luo, T. P. Pollard, S. Hou, T. Gao, X. Fan, C. Cui, J. Yue, Y. Tong, G. Yang, T. Deng, M. Zhang, J. Ma, L. Suo, O. Borodin, C. Wang, "A Pyrazine-Based Polymer for Fast-Charge Batteries" *Angew. Chemie - Int. Ed.* **2019**, *58*, 17820–17826.

[7] W. Luo, Y. Liu, Z. Zhang, F. Li, Z. Chao, J. C. Fan, "Rational Molecular Design Strategy of a Carbonyl Cathode for Better Aluminum Organic Batteries" *ACS Sustain. Chem. Eng.* **2023**, *11*, 11406–11414.

[8] X. Peng, A. Baktash, N. Alghamdi, M. M. Rana, Y. Huang, X. Hu, C. He, Z. Luo, J. Ning, L. Wang, B. Luo, "Boosting Aluminum Storage in Highly Stable Covalent Organic Frameworks with Abundant Accessible Carbonyl Groups" *Adv. Energy Mater.* **2024**, *14*, 2400147.

[9] Y. Wang, K. Long Ng, G. Azimi, "An Aluminum-Benzo[1,2-b:4,5-b']dithiophene-4,8-dione Organic Rechargeable Battery Featuring Low Self-Discharge" *Batter. Supercaps* **2022**, *5*, e202200182.

[10] L. Zhou, Z. Zhang, L. Cui, F. Xiong, Q. An, Z. Zhou, X. F. Yu, P. K. Chu, K. Zhang, "High-Capacity and Small-Polarization Aluminum Organic Batteries Based on Sustainable Quinone-Based Cathodes with $Al^{3+}$ Insertion" *Cell Rep. Phys. Sci.* **2021**, *2*, 100354.

[11] G. Wu, C. Lv, W. Lv, X. Li, W. Zhang, Z. Li, "Anthraquinone Derivatives Supported by $Ti_3C_2$(MXene) as Cathode Materials for Aluminum-Organic Batteries" *J. Energy Chem.* **2022**, *74*, 174–183.

[12] J. Bitenc, N. Lindahl, A. Vizintin, M. E. Abdelhamid, R. Dominko, P. Johansson, "Concept and Electrochemical Mechanism of an Al Metal Anode-Organic Cathode Battery" *Energy Storage Mater.* **2020**, *24*, 379–383.

[13] D. J. Kim, D. J. Yoo, M. T. Otley, A. Prokofjevs, C. Pezzato, M. Owczarek, S. J. Lee, J. W. Choi, J. F. Stoddart, "Rechargeable Aluminum Organic Batteries" *Nat. Energy* **2019**, *4*, 51–59.

[14] M. Mao, Z. Yu, Z. Lin, Y. S. Hu, H. Li, X. Huang, L. Chen, M. Liu, L. Suo, "Simplifying and Accelerating Kinetics Enabling Fast-Charge Al Batteries" *J. Mater. Chem. A* **2020**, *8*, 23834–23843.

[15] X. Peng, Y. Xie, A. Baktash, J. Tang, T. Lin, X. Huang, Y. Hu, Z. Jia, D. J. Searles, Y. Yamauchi, L.

Wang, B. Luo, "Heterocyclic Conjugated Polymer Nanoarchitectonics with Synergistic Redox-Active Sites for High-Performance Aluminum Organic Batteries" *Angew. Chemie - Int. Ed.* **2022**, *61*, e202203646.

[16] R. Grieco, O. Luzanin, D. Alvan, M. Liras, R. Dominko, N. Patil, J. Bitenc, R. Marcilla, "A Phenazine-Based Conjugated Microporous Polymer as a High-Performing Cathode for Aluminum-Organic Batteries" *Faraday Discuss.* **2023**, *250*, 110–128.

[17] K. Pirnat, J. Bitenc, A. Vizintin, A. Krajnc, E. Tchernychova, "Indirect Synthesis Route toward Cross-Coupled Polymers for High Voltage Organic Positive Electrodes" *Chem. Mater.* **2018**, *30*, 5726–5732.

[18] X. Wang, H. Dong, A. Eddine Lakraychi, Y. Zhang, X. Yang, H. Zheng, X. Han, X. Shan, C. He, Y. Yao, "Electrochemical Swelling Induced High Materials Utilization of Porous Polymers in Magnesium Electrolytes" *Mater. Today* **2022**, *55*, 29–36.

[19] A. Vizintin, J. Bitenc, A. Kopač Lautar, K. Pirnat, J. Grdadolnik, J. Stare, A. Randon-Vitanova, R. Dominko, "Probing Electrochemical Reaction in Organic Cathode Materials vis in Operando Infrared Spectroscopy" *Nat. Commun.* **2018**, *9*, 661.

[20] J. Bitenc, U. Košir, A. Vizintin, N. Lindahl, A. Krajnc, K. Pirnat, I. Jerman, R. Dominko, "Electrochemical Mechanism of Al Metal-Organic Battery Based on Phenanthrenequinone" *Energy Mater. Adv.* **2021**, *2021*, 9793209.
